# Deciphering the evolutionary signatures of pinnipeds using novel genome sequences: The first genomes of *Phoca largha*, *Callorhinus ursinus*, and *Eumetopias jubatus*

**DOI:** 10.1038/s41598-018-34758-0

**Published:** 2018-11-15

**Authors:** Jung Youn Park, Kwondo Kim, Hawsun Sohn, Hyun Woo Kim, Yong-Rock An, Jung-Ha Kang, Eun-Mi Kim, Woori Kwak, Chul Lee, DongAhn Yoo, Jaehoon Jung, Samsun Sung, Joon Yoon, Heebal Kim

**Affiliations:** 1 Biotechnology Research Division, National Institute of Fisheries Science, 216 Haean-ro, Gijang-eup, Gijang gun, Busan 46083 Republic of Korea; 2 Interdisciplinary Program in Bioinformatics, Seoul National University, Kwan-ak Gu, Seoul Republic of Korea; 3 C&K genomics, C-1008, H businesspark, 26, Beobwon-ro 9-gil, Songpa-gu, Seoul Republic of Korea; 4 Cetacean Research Institute, National Institute of Fisheries Science, 250 Jangsaengpo Gorae-ro, Nam-gu, Ulsan 44780 Republic of Korea; 5 Department of Taxonomy and Systematics, National Marine Biodiversity Institute of Korea, eocheon-gun, Chungcheongnam-do 33662 Republic of Korea; 6 Department of Agricultural Biotechnology, Seoul National University, Kwan-ak Gu, Seoul Republic of Korea

## Abstract

The pinnipeds, which comprise seals, sea lions, and walruses, are a remarkable group of marine animals with unique adaptations to semi-aquatic life. However, their genomes are poorly characterized. In this study, we sequenced and characterized the genomes of three pinnipeds (*Phoca largha*, *Callorhinus ursinus*, and *Eumetopias jubatus*), focusing on site-wise sequence changes. We detected rapidly evolving genes in pinniped lineages and substitutions unique to pinnipeds associated with amphibious sound perception. Phenotypic convergence-related sequence convergences are not common in marine mammals. For example, *FASN*, *KCNA5*, and *IL17RA* contain substitutions specific to pinnipeds, yet are potential candidates of phenotypic convergence (blubber, response to hypoxia, and immunity to pathogens) in all marine mammals. The outcomes of this study will provide insight into targets for future studies of convergent evolution or gene function.

## Introduction

Marine mammals are a classic example of convergent evolution in terms of adaptation of terrestrial mammals to the marine environment. During secondary adaptation to the marine environment, marine mammals experienced similar environmental challenges, which have resulted in shared morphological or physiological features across distant taxa. For instance, they have experienced similar changes in skin and limbs, and subsequently became streamlined^1,2^. Adaptive traits related to hypoxia are shared features of marine mammals^2,3^.

Marine mammals include three orders: cetaceans (whales, dolphins, and porpoises), pinnipeds (seals, sea lions, and walruses), and sirenians (manatees and dugongs)^4^. They have evolved to inhabit the ocean in multiple lineages. Cetaceans and sirenians emerged around 40–50 million years ago (mya) from Cetartiodactyla and Afrotheria, respectively^5^. Pinnipeds emerged within the Carnivora approximately 20 million years later^5^. This implies that different molecular changes occurred across separate lineages, possibly resulting in divergent phenotypic changes. However, most studies related to marine mammals have focused on convergent evolution, although some of the adaptations of marine mammals to an aquatic lifestyle vary among species^5^.

Pinnipeds, which consist of three families (*Phocidae*, *Otariidae*, and *Odobenidae*) are distinguishable from other marine mammals^6^. Most pinnipeds are semi-aquatic, unlike other marine mammals that spend their entire lives in the water^4^, and have modified limbs as flippers that propel them both in the water and on land^7^. In addition, with the exception of the walrus, which is the only extant species of the family *Odobenidae*, all pinnipeds have fur coats^8^. These distinct characteristics have not been sufficiently characterized at the molecular level. Although a draft fur seal genome has recently been assembled^9^, the evolutionary and biological aspects of pinnipeds have not been investigated. Indeed, the genome of the Weddell seal (family *Phocidae*) has not been completed (http://software.broadinstitute.org/allpaths-lg/blog/?p=647). In addition, most phylogenetic studies of pinnipeds have used limited marker sequences, such as that of the mitochondrial genome^10–12^.

Comparative genomics enables investigation of the convergent evolution of distant species. For example, convergent amino acid changes for vocal learning were identified by sequencing 48 avian genomes^13^. Similarly, Parker *et al*.^14^ reported nearly 200 convergent loci in the genomes of echolocating mammals. Although there are more studies to demonstrate to phenotypic convergence-linked sequence convergence, molecular convergence toward phenotypic convergence, at least in marine mammals, seems to be uncommon. By analyzing 22 mammalian genomes, including those of three marine mammals, Foote *et al*.^15^ suggested that different molecular pathways could be used to reach the same phenotype. In a study of the *Hox* gene family in mammals, only a fraction of sites had positive selection signatures shared by three independent marine mammal lineages^16^. Rather than sequence-level, gene-level convergence was presented as widespread signatures when evolutionary rates were used^2^. Therefore, there is convergence at the functional level or higher in separate mammalian lineages, and different marine mammal lineages have used different molecular pathways to achieve phenotypic convergence.

Here, we constructed draft genomes of three species of two pinniped families: *Phoca largha* (*Phocidae*) and *Callorhinus ursinus* and *Eumetopias jubatus* (*Otariidae*) (Fig. S1 and Supplementary Note S1). We identified genes with a positive selection signature that were common to the three pinnipeds but absent from other mammals, which are likely related to the unique traits of pinnipeds. In addition, divergent molecular changes likely to occur only in the pinniped lineage during phenotypic convergence of marine mammals were investigated.

## Results

### Genome assembly and annotation

Before assembling the genomes of the three pinnipeds, we estimated the genome sizes using the 19-mer distribution of paired-end reads. The estimated genome sizes were 2.61, 2.71, and 2.64 Gbp for the spotted seal (SS), northern fur seal (NFS), and Steller sea lion (SSL), respectively (Fig. S2). The genomic DNA of the three pinnipeds was assembled to a size of approximately 2.5 Gbp, which is similar to that of previously assembled genomes (Antarctic fur seal^9^, Hawaiian monk seal [https://www.ncbi.nlm.nih.gov/assembly/GCF_002201575.1], and Weddell seal [https://www.ncbi.nlm.nih.gov/assembly/GCF_000349705.1]). Summary statistics of the final assembly are provided in Table S1. To assess the quality of the draft genomes, we remapped paired-end reads with a 350 bp insert size, which yielded alignment rates of >98% for the three genomes (98.24, 98.74, and 98.73% for SS, NFS, and SSL, respectively). The completeness of core-orthologs was evaluated using Benchmarking Universal Single-Copy Orthologs (BUSCO). Each of the three genomes contained more than 90% core-orthologs from the class Mammalia, in the form of either complete or fragmented sequences (Table S2). The GC contents of the three genomes were investigated using 500 bp bins, and were similar to those of the draft genomes of related species (Fig. S3).

Repeat elements accounted for 35.83, 40.40, and 35.78% of the SS, NFS, and SSL genomes, respectively. Of the repeat regions, long interspersed nuclear element (LINE) was the most extended element in terms of base pairs (Table S3). After masking the identified repeat elements, 33,988, 32,740, and 28,081 protein-coding genes were predicted for SS, NFS, and SSL, respectively (Table S4). Of the predicted genes, ~95% were functionally annotated to at least one of the InterPro, SwissProt, and TrEMBL databases (Table S5).

Therefore, the SS, NFS, and SSL genomes were not significantly different from one another in terms of various statistics related to genome assembly. Because the three species are related, this similarity suggests that the three genomes have similar levels of completeness.

### Phylogenomics and protein-coding gene families

**Figure 1:**
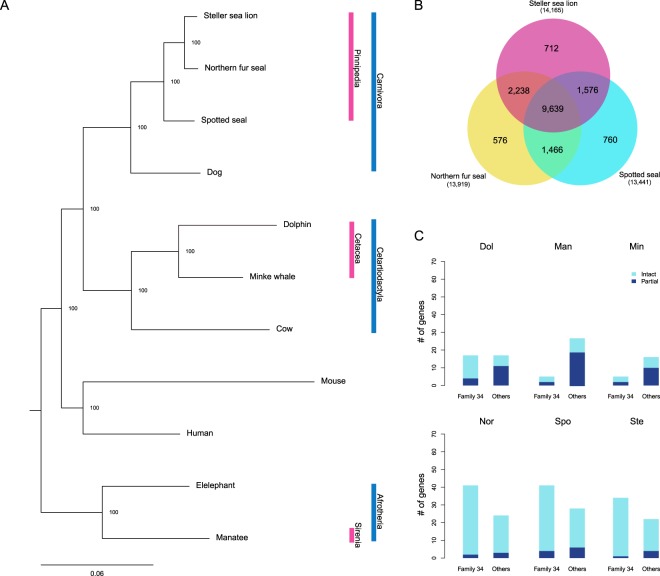
Phylogenomics and protein-coding gene families of pinnipeds. (**A**) Species tree of 12 terrestrial and marine mammals constructed by the maximum-likelihood method. (**B**) Orthologous gene clusters in three pinnipeds. (**C**) Number of intact (coverage ≥ 90%) and partial (coverage < 90%) genes that belong to Protocadherin gene families, named family 34 in our dataset (Dol, dolphin; Man, manatee; Min, Minke whale; Nor, northern fur seal; Spo, spotted seal; Ste, Steller sea lion).

To identify the relationships among SS, NFS, and SSL and other related species, we constructed a maximum-likelihood (ML) tree using the amino acid sequence of one-to-one orthologs generated using a dataset of the proteomes of nine species available in public databases. In total, there were 2,907 one-to-one orthologs, the combined length of which was 982,250 amino acid residues. The newly constructed tree provided robust support for the known phylogenetic tree of marine mammals (http://www.timetree.org/) (Fig. 1A), and the phylogenetic tree is used in the downstream analysis for positively selected genes and substitutions.

We constructed orthologous gene clusters using the genomes of six marine mammals to identify gene clusters and their functions unique to pinnipeds (Fig. S4). The pinniped genomes contained 13,919 (NFS), 13,441 (SS), and 14,165 (SSL) orthologous gene families, respectively, 9,639 of which were shared by all three pinnipeds (Fig. 1B). Of these gene families, 1,874 were present in all pinnipeds, but not in three other mammals. By Gene Ontology (GO) enrichment analysis, we found these gene families to be enriched in 31 terms (p-value < 0.05), several of which were related to an aquatic lifestyle, such as ‘aorta development’, ‘sterol biosynthetic process’, ‘cardiac septum development’, ‘coronary vasculature development’, and ‘cellular response to oxidative stress’ (Table S6).

To investigate gene-family expansion and contraction, a computational analysis of gene-family sizes using the orthologous gene clusters was performed in CAFÉ^17^. By comparing six marine mammals, we found that 874 gene families were expanded, while 1,925 gene families were contracted in the pinniped lineage. Of these gene families, a subset of the Protocadherin (Pcdh) family (herein named family 34) was significantly expanded in the pinniped lineage (p = 0.000346). The genomes of the pinnipeds contained a larger number of Pcdh genes than those of the other marine mammals (Fig. 1C). Pcdhs are the largest mammalian subgroup of the cadherin superfamily^18^, and have functions associated with the nervous system^19,20^ such as in olfactory sensory neurons^21^. The number of Pcdhs varies among vertebrate lineages^22^.

### Genes with accelerated evolution in the pinniped lineage

To detect positive selection in the pinniped lineage, a dN/dS analysis using the branch-site model was performed. The branch-site model allows dN/dS (ω) to vary both among sites in the protein and across branches on the tree^23^. Therefore, we hypothesized a few sites in the pinniped branches to have different ω ratios compared to other branches and that the genes containing these sites might be related to the unique features of pinnipeds. After the filtering step (see Methods), we analyzed 2,754 one-to-one orthologs identified in the proteomes of 12 mammals, of which seven genes with 145 sites were under positive selection (Bonferroni-corrected p < 0.05, posterior probability based on Bayes empirical Bayes inference [BEB] >0.95; Table 1). Of these genes, transmembrane protein 132B (*TMEM132B*) contained the largest number of positively selected sites (52 sites). Of the seven genes, six contained 29 conserved domains with 74 sites (51%) under positive selection. GO terms were assigned to each gene, and the following functional associations with pinniped lifestyle were found: *TECTA*, sensory perception of sound (GO:0007605), *SPEG*, muscle organ development (GO:0007517), and *ADAMTS5*, defence response to bacterium (GO:0042742) and tooth eruption (GO:0044691). *TECTA* encodes alpha-tectorin, a major non-collagenous glycoprotein of the tectorial membrane, an extracellular matrix in the inner ear^18^. Mutations in *TECTA* result in hearing loss^24–26^ (OMIM: 602574). *SPEG* is required for cardiac development and is associated with cardiac myopathy^27,28^ (OMIM: 615950). *ADAMTS5*, which encodes an extracellular matrix-degrading enzyme, plays an important role in the T-cell immune response to viral infection^29,30^.

**Table 1:** Genes with accelerated evolution in the pinniped lineage. H1_fg_omega: dN/dS value (ω) on foreground given H_1_ (ω varies across the branches); H0_lnl: log likelihood given H_0_ (ω does not vary across the branches); H1_lnl: log likelihood given H_1_; H0_lnl: log likelihood given H_0_.

Gene	H1_fg_omega (ω_2_)	Proportion (H_1_) (1 – p_0_ – p_1_)p_1_/(p0 + p_1_)	H0_lnl	H1_lnl	Likelihood ratio	p-value	Adjusted p-value	# of positively selected sites*
*TMEM132B*	3.81581	0.01666	−6438.78	−6419.68	38.20475	6.37E-10	1.18E-06	52 (22)
*PARP1*	4.76894	0.00604	−5357.53	−5341.29	32.48145	1.20E-08	2.22E-05	23 (22)
*TECTA*	3.67139	0.00194	−12076.1	−12060.4	31.42787	2.07E-08	3.83E-05	18 (14)
*FUBP3*	4.89809	0.01916	−4880.95	−4869.76	22.38143	2.24E-06	0.004144	12 (1)
*IGF2BP1*	4.96893	0.00201	−4448.2	−4438.13	20.13898	7.20E-06	0.01332	19 (2)
*SPEG*	4.81594	0.00254	−11218.8	−11209.4	18.85029	1.41E-05	0.026085	13 (13)
*ADAMTS5*	4.38148	0.00124	−4320.48	−4311.43	18.1014	2.09E-05	0.038665	8 (0)

^*^Number of positively selected sites with a BEB of **>**0.95. The numbers of positively selected sites within domain regions are shown in parentheses.

**Figure 2:**
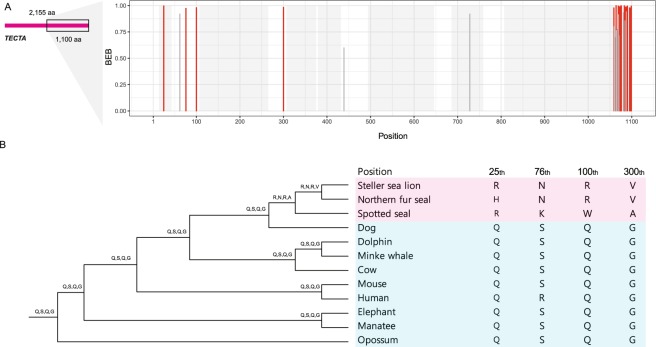
Results of a branch-site model analysis of *TECTA*. (**A**) Bayes empirical Bayes (BEB) posterior probability in *TECTA*. Shaded area, conserved domain regions. (**B**) Sequence of sites with significant BEB (>0.95). Red and blue shaded areas, pinnipeds and other mammals, respectively.

To assess their uniqueness, the amino acid residues positively selected in the pinniped lineage were compared to other species in our analysis as well as in publicly available databases. For example, we investigated 4 of the 18 sites within *TECTA* after manually filtering out amino acid residues with spurious alignment (Fig. 2A). The four sites were pinniped-specific compared to the other nine species (Fig. 2B). Moreover, a 100-way multi-alignment showed that two pinnipeds (Pacific walrus and Weddell seal) had residues identical to those in the three pinnipeds in this study (Fig. S5). We could only find a small number of residues matching those in 100 vertebrates at these sites (Fig. S5). Consequently, the four sites within *TECTA* might be unique to pinnipeds and generated during their adaptation to a semi-aquatic environment.

### Unique substitutions of pinnipeds contributed to the phenotypic convergence of marine mammals

Parallel substitutions are widespread in marine mammals; however, most are not unique to marine mammals^15,31^. Moreover, molecular convergences are rarely linked to phenotypic convergences in marine mammals^2,15,16^. In this study, about half of the parallel substitutions shared by marine mammals were also found in terrestrial mammals, and a considerable number of unique substitutions was found between species with no obvious phenotypic convergence (Figs S6–S8). Therefore, we hypothesized the existence of pinniped-specific substitutions that contributed to aquatic adaptation and are shared by marine mammals.

First, we focused on gene-level convergence (Fig. S9) and conducted a dN/dS analysis of one-to-one orthologs using the branch model. The branch model allows the dN/dS (ω) ratio to vary among branches in the phylogeny and is useful for detecting positive selection acting on particular lineages^32^. In this way we aimed to detect candidate genes with different ω ratios among the marine mammal lineages rather than candidate sites, which may contribute to phenotypic convergence among marine mammals. Of the 2,754 filtered one-to-one orthologs, the branch model-based dN/dS analysis detected 853 positively selected genes in marine mammal lineages (Fig. S10b, cetaceans, pinnipeds, and sirenians, Bonferroni corrected p-value < 0.05). These are hereafter referred to as rapidly evolving genes (REGs). A subset of 853 REGs covered the following functional categories possibly associated with marine mammals’ adaptation to the ocean: muscle physiology (GO:0007015, GO:0035914, GO:0007519, and GO:0035914), lipid metabolism (GO:0006629, GO:0006869, GO:0006631, and GO:0016042), sensory system (GO:0007605, GO:0042472, and GO:0021772), skin and connective tissue (GO:0008544, GO:0043588, and GO:0030216), cardiovascular system (GO:0086091, GO:0060976, and GO:0007507), and resistance to oxidative stress (GO:0001666).

We also calculated the site-wise log likelihood support (SSLS) values for the amino acid sequences of 2,754 genes (996,522 residues in total) and calculated the ΔSSLS values to detect site-wise signatures of divergent evolution. The ΔSSLS value is indicative of the goodness-of-fit of each site to a pair of phylogenetic trees. We aimed to detect genes positively selected in three marine mammal lineages with substitutions unique to pinnipeds. Therefore, we calculated the SSLS for two hypotheses: H_0_, divergence among marine mammal clades and H_1_, convergence among marine mammal clades. Therefore, a ΔSSLS (log likelihood of H_0_ − log likelihood of H_1_) value > 0 means that the site in question supports divergence among marine mammal clades. We used the ΔSSLS value as a filtering criterion to exclude sites supporting convergence among marine mammals. By excluding those with low ΔSSLS values, we identified pinniped-specific sites that support the separation clades of marine mammals. We expected that this analysis would generate more reliable sites than directly extracting unique substitutions over REGs, as it considers the overall phylogeny not just the sequence itself.

**Figure 3:**
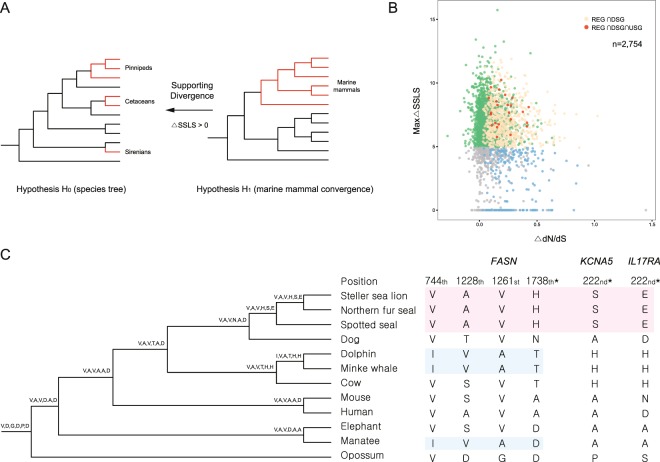
Analysis of rapidly evolving genes, divergent substitution genes, and unique substitution genes. (**A**) Hypotheses used to calculate ΔSSLS. (**B**) ΔdN/dS and ΔSSLS distribution in 2,754 orthologs. (**C**) Unique substitutions of *FASN*, *KCNA5*, and *IL17RA*. Asterisks, substitutions unique to pinnipeds. Other positions represent substitutions unique to cetaceans + sirenians.

We regarded the 9,965 residues with the top 1% ΔSSLS values as being supported by divergent substitutions (support for H_0_) rather than convergent substitutions among three marine mammal clades (support for H_1_) (Fig. 3A). We termed the 2,159 genes containing at least one of these residues as divergent substitution genes (DSGs). DSGs covered most of the 2,754 one-to-one orthologs (78%), and 85% of total residues had positive ΔSSLS values. Therefore, the majority of the sequences supported the commonly accepted phylogeny.

Of the 853 REGs, 658 (3,277 residues) had a least one top 1% ΔSSLS site (Fig. 3B). Although these genes covered the functional categories associated with marine mammals’ adaptation, a single residue divergence supported by likelihood divergence (ΔSSLS) could be vulnerable to sequencing error. We also focused on sequence changes common to the pinniped clade; that is, changes from the ancestral node sequence shared by dog to that of the node of each pinniped. Therefore, we investigated unique substitutions (any amino acid residue at the same position in all three pinnipeds that was found in neither the ancestral nodes with their respective terrestrial taxa [dog] nor in other mammals) to rule out sequence divergences other than ancestral substitutions unique to the pinniped clade. There were 1,731 genes with at least one unique substitution (7,878 residues); these were termed unique substitution genes (USGs), 63 of which contained top 1% ΔSSLS residues at the same positions as unique substitutions. Finally, we obtained 24 REGs containing top 1% ΔSSLS residues and unique substitutions at the same positions (Fig. 3 and Table 2).

**Table 2:** Genes with sequence changes likely to occur in only the pinniped lineage when gene-level convergence took place in marine mammals.

Gene	H1_fg_omega	H0_lnl	H1_lnl	p-value	Adjusted p-value	Max ΔSSLS	# of unique substitutions
*VPS45*	0.40038	−4037.348759	−3956.066722	3.11E-37	8.3037E-34	7.730292	1
*ABCC10*	0.44339	−18216.8972	−18153.02735	1.28E-29	3.4176E-26	8.828157	20
*FASN*	0.19743	−40595.23443	−40538.33849	1.45E-26	3.8715E-23	9.661292	54
*DUS3L*	0.34814	−8646.525061	−8591.484612	9.41E-26	2.51247E-22	8.224026	5
*DDAH2*	0.45032	−2977.374327	−2929.688683	1.58E-22	4.2186E-19	6.582644	3
*SASH1*	0.19451	−6515.324513	−6472.937933	3.35E-20	8.9445E-17	6.525431	6
*GPR155*	0.57001	−5926.033389	−5888.825926	6.33E-18	1.69011E-14	6.877673	6
*DUSP27*	0.28847	−13197.13408	−13162.41783	7.91E-17	2.11197E-13	6.749015	79
*EMILIN3*	0.26942	−9364.829765	−9346.305662	1.15E-09	3.0705E-06	8.265838	11
*DCLRE1A*	0.70785	−6950.035862	−6931.672364	1.36E-09	3.6312E-06	8.100957	6
*DGKQ*	0.1879	−12994.3629	−12976.13466	1.56E-09	4.1652E-06	7.537842	13
*VWF*	0.21584	−26711.98598	−26695.2834	7.48E-09	1.99716E-05	8.820315	34
*GUCY2C*	0.45014	−6447.80369	−6431.307957	9.26E-09	2.47242E-05	5.926296	4
*ABCD4*	0.23237	−6428.830395	−6414.05014	5.42E-08	0.000144714	6.854527	7
*TACC3*	0.48752	−7423.970534	−7410.509676	0.000000212	0.00056604	8.265423	6
*LMTK2*	0.34446	−19363.6547	−19351.05935	0.000000519	0.00138573	8.54542	12
*RIN3*	0.27223	−6861.555137	−6849.333436	0.000000765	0.00204255	5.750655	6
*KCNA5*	0.18524	−6876.444906	−6864.677432	0.00000123	0.0032841	6.664093	6
*TRMT12*	0.48457	−6214.487554	−6203.00578	0.00000165	0.0044055	7.141302	7
*POLL*	0.41568	−7373.848752	−7362.382731	0.00000168	0.0044856	9.073179	9
*ANKRD5*	0.3288	−9488.47528	−9477.525821	0.00000287	0.0076629	9.485991	10
*LAMB2*	0.23606	−17812.14129	−17801.84156	0.00000566	0.0151122	7.663766	8
*IL17RA*	0.40977	−10647.044	−10636.87528	0.00000649	0.0173283	10.242048	12
*TRIML1*	0.45219	−4898.490822	−4888.744854	0.0000101	0.026967	7.674387	4

H1_fg_omega: dN/dS value (ω) on foreground branches given H_1_ (ω varies across the branches); H0_lnl: log likelihood given H_0_ (ω does not vary across the branches); H1_lnl: log likelihood given H_1_.

Although the 24 REGs are supported by rapid evolutionary rates (dN/dS) and fixation of amino acid residues within the pinniped clade, the precise phenotypic effects of the unique substitutions cannot currently be ascertained. However, several of the 24 REGs have known functional associations that suggest a role in the convergent phenotypic evolution of marine mammal lineages. For instance, *FASN* encodes fatty-acid synthase, which catalyzes the conversion of acetyl-CoA and malonyl-CoA to long-chain saturated fatty acids^33^ and is related to obesity^34^. *KCNA5* (potassium voltage-gated channel subfamily A member 5) encodes voltage-gated potassium channels in pulmonary artery smooth muscle cells and mediates the response to hypoxia^35,36^. *IL17RA* encodes the interleukin 17 A receptor, a ubiquitous type I membrane glycoprotein that binds to interleukin 17 A. Interleukin 17 A and its receptor play a key role in the immune response to pathogen infection^37,38^.

## Discussion

In this study, we presented three genomes of pinnipeds (*Phoca largha*, *Callorhinus ursinus* and *Eumetopias jubatus*) that belong to Phocidae, and Otariidae family for the first time. Pinnipedia is a monophyletic group distinct from other marine mammals in many respects, such as its semi-aquatic lifestyle and well-developed flippers^5^. Our findings provide insight into the common features of pinniped genomes, which is less clear than the convergent evolution of pinnipeds.

Pinnipeds are the most amphibious mammalian species. Possibly, for that reason, their auditory systems are challenged by the need to function efficiently underwater and in air, unlike the solely underwater hearing of cetaceans and sirenians^39,40^. *TECTA*, which is related to sound perception^26^, was identified as positively selected in the pinniped lineage. *TECTA* encodes α-tectorin, a non-collagenous component of the tectorial membrane in the cochlea^41^. The tectorial membrane is an extracellular matrix that covers the surface of the sensory epithelium in the cochlea and plays a vital role in transmitting sound to the stereocilia of hair cells, where the sound is transduced into neural signals^42^. Therefore, mutations in *TECTA* might be involved in the semi-aquatic adaptation of pinnipeds by tuning their hearing ranges. Indeed, mutations in *TECTA* are responsible for loss of hearing at particular frequencies^43–45^. Interestingly, the four positively selected sites in *TECTA* were very rare among 100 vertebrates (Fig. S5). Although its relationship with amphibious sound perception is unclear, *TECTA* should be investigated in future studies of amphibious sound perception in pinnipeds. The pinniped lifestyle might influence the function of other candidate genes, such as *SPEG* and *ADAMTS5*. Comparative analysis of amphibious mammals may reveal their adaptations at the molecular level and show that an amphibious lifestyle results in selection pressure.

We found that a considerable number of parallel substitutions are not unique to marine mammals, consistent with two recent reports^15,31^. This implies that molecular convergence is not a driving force of phenotypic convergence among marine mammals, and that different clades of marine mammals used different molecular pathways to reach similar phenotypes. Although this phenomenon has been observed several times in marine mammals, whether it also applies to other clades is unclear. More evidence in other clades is needed to generalize this phenomenon to other forms of phenotypic convergence.

Because sequence convergences leading to phenotypic convergences are not common, we assumed that unique substitutions contributed to the aquatic adaptation of pinnipeds. In our analyses, three genes, *FASN*, *KCNA5*, and *IL17RA*, were identified as candidates. The well-defined roles of these genes (blubber^46^, resistance to hypoxia^47^, and the immune response to pathogens^15^, respectively) support their contributions to phenotypic convergences of marine mammals. *FASN* and *KCNA5* were not found to be positively selected in the branch-site model analysis using all marine mammal branches as foreground branches. In addition, only ~17% of the REGs were found to be positively selected genes by the branch-site model analysis (Fig. S11). Such results suggest that rapid evolution occurred at different sites of the candidate genes between marine mammal clades, an example of gene-level convergent evolution.

Convergent evolution can occur at molecule, gene, and function levels^31,48^. We focused on convergence at the gene level. However, the functions of the majority of the putative convergent genes were unrelated to apparent phenotypic convergence, such as lipid metabolism and resistance to oxidative stress. This may be due to the missing link between convergent genes and phenotypic convergences. In this case, the results can be complemented by studying the gene functions and convergence at a higher-level.

## Conclusions

We report here the genomes of *Phoca largha*, *Callorhinus ursinus*, and *Eumetopias jubatus*. These genomes suggest the existence of considerable sequence diversity within and across marine mammal species. We identified several unique genome-level adaptations to the semi-aquatic lifestyle of pinnipeds, and several examples for evolution of marine mammals that are convergent in gene-level, but divergent in sequence-level. These findings suggest targets for future *in vitro* and *in vivo* studies of adaptive phenotypes and provide insight into convergent evolution at the molecular level.

## Methods

### Ethics statement

No ethics approval was required for the collection of DNA from blood samples of bycaught carcasses.

### Sample information and collection

We collected five pinniped samples from Korean waters. Three male Northern fur seals (*Callorhinus ursinus*) were bycaught in set nets and collected during January and February 2016 (one was used to produce sequence data). A bycaught female Steller sea lion (*Eumetopias jubatus*) was collected in April 2008. A female spotted seal (*Phoca largha*) was collected on a beach in August 2015. All of the above were found in the waters off Gangwon-do, northeastern South Korea.

### DNA sequencing and genome assembly

For whole-genome shotgun sequencing and draft genome assembly, we constructed two paired-end libraries with insert sizes of 350 and 700 bp using the Illumina TruSeq DNA Sample Preparation Kit (Illumina, San Diego, CA, USA). For the Steller sea lion genome, mate-pair libraries with insert sizes of 3, 9, and 40 kb were constructed as scaffolds using the Illumina Nextera mate-pair library construction protocol (Illumina). Sequence reads were generated using the Illumina Nextseq 500 platform. Information on the constructed libraries and sequencing data is provided in Table S7.

The 19-mer distribution of the paired-end library with an insert size of 350 bp was calculated using Jellyfish^49^, and the sizes of three genomes were estimated (Fig. S1). To retrieve high-quality sequence reads, the quality of the raw data was controlled using FASTQC^50^. Artifact sequences were removed via Trimmomatic^51^ for paired-end libraries, and Nxtrim^52^ for mate-pair libraries. Sequencing errors within each read were estimated and discarded using the error-correction module of Allpaths-LG^53^. We assembled error-corrected paired-end reads using IDBA_UD^54^ with the option of pre-correction and kmin = 40. Scaffolding on initial contigs was conducted using the paired-end reads with a 700 bp insert size, and mate-pair reads sequentially by SSPACE^55^ and ScaffMatch^56^. After scaffolding, we iteratively filled gaps using Gapcloser^57^ with the -l 155 and -p 31 parameters.

RepeatModeler^58^, which includes RECON^59^, RepeatScout^60^, and TRF^61^, was used to create a custom database for each species. A custom library was constructed by integrating the custom databases into the Repbase^62^ database of mammals. Repeat elements were identified and masked using RepeatMasker^63^ with the custom library and ‘-q, no_is’ options.

### Genome annotation

Two approaches were used to predict protein-coding genes. First, manually curated protein sequences of Mammalia were retrieved from Swiss-Prot^64^ and aligned to the pinniped genomes using tBLASTn^65^. The homologous genome sequences with E-values ≤ 1E-5 were extracted and realigned to the matched proteins using Exonerate^66^ to predict splice sites. *Ab initio* gene prediction was conducted using Augustus^67^, Geneid^68^, and GlimmerHMM^69^ software with the default options. Predicted genes using each approach were combined using EvidenceModeler^70^ into a consensus gene set.

For assessment of the quality of the draft genome, we remapped paired-end reads with a 350 bp insert size and investigated completeness of core-orthologs using BUSCO^71^.

For the three gene sets, the best match of a BLASTP^72^ search against the SwissProt and TrEMBL databases^73^ was assigned to putative functions. Gene motifs and domains were determined using InterProScan v. 5.19^74^. The GO IDs for each gene were obtained from the corresponding InterPro entries.

### Ortholog identification

The complete proteome datasets were downloaded from UCSC Genome Browser^75^ for the following nine mammals: human (hg19), mouse (mm10), dog (canFam3), cow (bosTau8), manatee (triMan1), dolphin (turTru2), Minke whale (balAcu1), opossum (monDom5), and elephant (loxAfr3). Gene clusters for these nine mammals and three pinnipeds were identified using OrthoMCL v. 2.0.9^76^ with the default settings. A custom python script was used to generate a dataset comprising strict one-to-one orthologs (core-orthologs) from the 12 mammals.

### Phylogenomic analyses using a genome-wide set of one-to-one orthologs

Amino acid sequences of 12 mammals corresponding to the one-to-one orthologs were individually aligned using ClustalW v. 2.1^77^. A concatenated alignment was then prepared by merging individual alignments. The concatenated alignment was trimmed using Gblocks v. 0.91b^78^ with auto settings.

The best-fit substitution model for the alignment was determined using ModelGenerator^79^. For phylogenetic analyses, RAxML v. 7.2.8^80^ was used to generate ML trees. Rapid bootstrap analysis and identification of the best-scoring ML tree (-f a option) were performed using RAxML v. 7.2.8^80^. Bootstrap support values/percentages were determined using 100 replicates. A Jones-Taylor-Thornton amino acid substitution model^81^ (with the PROTCATIJTTF option) as recommended by ModelGenerator^82^ was used to construct the ML trees.

### Detection of lineage-specific gene losses and gains

Using the gene clusters defined by Orthomcl v. 2.0.9^76^, the genes in each gene family group were enumerated and converted to input data for CAFÉ software v. 3.1^17^. Expansion or contraction of the gene families was defined by comparing the cluster size of the ancestor to that of each of the current species using CAFÉ^17^.

### Detection of positively selected genes and substitutions

To detect positively selected genes, coding sequence alignments were prepared by pal2nal v. 14^83^ using the amino acid alignments of the one-to-one orthologs. After trimming of the poorly aligned regions, alignments that are shorter than 100 bp or contain an internal stop codon were excluded.

To detect positive selection affecting a few sites in particular lineages (foreground branches, pinniped lineage in this study), we employed a branch-site model, which allows the ω ratio to vary both among lineages and among sites. We used the ML method of codeml in PAML v. 4.9^84^, which estimates the rate of non-synonymous substitutions (dN), the rate of synonymous substitutions (dS), and the ratio of the non-synonymous to synonymous substitution rates (ω) values using the F3X4 codon frequencies. An alternative codon substitution model was specified using model = 2, NSsites = 2 (model A^23,85^, number of parameters k = 4), which was compared with the corresponding null model ω_2_ = 1 (ω ratio of foreground branches) fixed (fix_omega = 1 and omega = 1) using a likelihood-ratio test (LRT). From the alternative model, two different ω ratios of site class 2b (proportion: (1 – p_0_ – p_1_) p_1_/(p_0_ + p_1_), ω_1_ = 1, ω_2_ ≥ 1) for pinniped branches (foreground branches) and other branches (background branches) were estimated (Fig. S10a) to detect positive selection.

To identify fast-evolving genes in marine mammals (pinnipeds, cetaceans, and sirenians), we employed a branch model, which allows the ω ratio to vary among branches^32^. In codeml, an alternative codon substitution model was specified using model = 2 and NSistes = 0, which was compared with the basic null model (model = 0, NSsites = 0) by LRT. From the alternative model, two different ω ratios for marine mammal branches (foreground branches) and other branches (background branches) were estimated (Fig. S10b).

Genes with a maximum dS of >3 or maximum dN/dS of >5 in all branches or a log-likelihood ratio of <0 were filtered from the output of each analysis. The Bonferroni method^46^ was used to correct for multiple testing, and a value of p < 0.05 was taken to indicate statistical significance.

### Calculation of site-wise likelihood support

To detect sites with molecular divergence that supported the monophyly of pinnipeds, we fitted the amino acid sequence alignment of one-to-one orthologs to a null model (H_0_, species tree) and an alternative model (H_1_, monophyly of marine mammals) (Fig. 3A). The goodness-of-fit of each site to a pair of phylogenetic trees under a given model was calculated as the SSLS value and directly compared as ΔSSLS = lnL (H_0_) - lnL (H_1_). Positive ΔSSLS values indicate a better fit of the model to the species tree, H_0_ (supporting divergence), whereas negative ΔSSLS values indicate a better fit to H_1_ (supporting convergence). The substitution model for each gene was determined by ModelGenerator^82^. The SSLS value for each site of alignment was estimated by RAxML v. 7.2.8^80^.

### Identification of parallel and unique substitutions

We defined parallel substitutions as any amino acid change at the same position in marine mammals different from that of the ancestral node of each marine group, but identical in the three marine groups. To identify parallel amino acid changes in marine mammals, the species tree constructed in this study was used to reconstruct the ancestral sequences. The ancestral sequences for each node were reconstructed by Joint method using FastML v. 3.1^86^. We allowed FastML 3.1 to estimate the branch length of the phylogenetic tree for each gene when the ancestral sequences were reconstructed using the set of 12 mammals. For the sites with parallel and unique substitutions, the amino acid sequences of 100 vertebrates were investigated by 100-way multi-alignment^87^ with the UCSC genome browser.

### Conserved domain search

To determine whether positively selected sites are located in gene functional domains, we searched for conserved domains within positively selected genes using the CD-Search tool in the NCBI^88^. The amino acid sequences of human orthologs were used as a query set with the following settings: data source, CDD v. 3.16; expected value threshold, 0.01; composition-based statistical adjustment, applied; low-complexity filter, not applied.

### Gene ontology analysis

We mapped the identified genes to GO categories in Ensembl^89^ to identify those putatively associated with a specific function, such as adipose tissue development. Gene set enrichment tests were performed by DAVID functional annotation^90^ using a cutoff P-value of <0.05.

## Electronic supplementary material

Supplementary information

## Data Availability

The datasets generated during the current study are available in the NCBI repository, PRJNA422019.

## References

[1] Fish FE, Howle LE, Murray MM (2008). Hydrodynamic flow control in marine mammals. Integrative and Comparative Biology.

[2] Chikina M, Robinson JD, Clark NL (2016). Hundreds of genes experienced convergent shifts in selective pressure in marine mammals. Molecular biology and evolution.

[3] Andersen HT (1966). Physiological adaptations in diving vertebrates. Physiological Reviews.

[4] Jefferson, T. A., Leatherwood, S. & Webber, M. A. *Marine mammals of the world*. (Food & Agriculture Org. 1993).

[5] Berta, A., Sumich, J. L. & Kovacs, K. M. *Marine mammals: evolutionary biology*. (Academic Press 2005).

[6] Berta A (2002). Pinnipedia, overview. J. Zool.

[7] Rybczynski N, Dawson MR, Tedford RH (2009). A semi-aquatic Arctic mammalian carnivore from the Miocene epoch and origin of Pinnipedia. Nature.

[8] Riedman, M. *The pinnipeds: seals*, *sea lions*, *and walruses*. (Univ of California Press 1990).

[9] Humble E (2016). A draft fur seal genome provides insights into factors affecting SNP validation and how to mitigate them. Molecular ecology resources.

[10] Slade RW, Moritz C, Heideman A (1994). Multiple nuclear-gene phylogenies: application to pinnipeds and comparison with a mitochondrial DNA gene phylogeny. Molecular Biology and Evolution.

[11] Davis CS, Delisle I, Stirling I, Siniff DB, Strobeck C (2004). A phylogeny of the extant Phocidae inferred from complete mitochondrial DNA coding regions. Molecular phylogenetics and evolution.

[12] Fulton TL, Strobeck C (2010). Multiple markers and multiple individuals refine true seal phylogeny and bring molecules and morphology back in line. Proceedings of the Royal Society of London B: Biological Sciences.

[13] Zhang G (2014). Comparative genomics reveals insights into avian genome evolution and adaptation. Science.

[14] Parker Joe, Tsagkogeorga Georgia, Cotton James A., Liu Yuan, Provero Paolo, Stupka Elia, Rossiter Stephen J. (2013). Genome-wide signatures of convergent evolution in echolocating mammals. Nature.

[15] Foote AD (2015). Convergent evolution of the genomes of marine mammals. Nature genetics.

[16] Nery MF, Borges B, Dragalzew AC, Kohlsdorf T (2016). Selection on different genes with equivalent functions: the convergence story told by Hox genes along the evolution of aquatic mammalian lineages. BMC evolutionary biology.

[17] De Bie T, Cristianini N, Demuth JP, Hahn MW (2006). CAFE: a computational tool for the study of gene family evolution. Bioinformatics.

[18] Hulpiau P, Van Roy F (2009). Molecular evolution of the cadherin superfamily. The international journal of biochemistry & cell biology.

[19] Wang X (2002). Gamma protocadherins are required for survival of spinal interneurons. Neuron.

[20] Chen WV (2012). Functional significance of isoform diversification in the protocadherin gamma gene cluster. Neuron.

[21] Hasegawa S (2008). The protocadherin-α family is involved in axonal coalescence of olfactory sensory neurons into glomeruli of the olfactory bulb in mouse. Molecular and Cellular Neuroscience.

[22] Yagi Takeshi (2008). Clustered protocadherin family. Development, Growth & Differentiation.

[23] Yang Z, Wong WS, Nielsen R (2005). Bayes empirical Bayes inference of amino acid sites under positive selection. Molecular biology and evolution.

[24] Collin Rob W J, de Heer Anne-Martine R, Oostrik Jaap, Pauw Robert-Jan, Plantinga Rutger F, Huygen Patrick L, Admiraal Ronald, de Brouwer Arjan P M, Strom Tim M, Cremers Cor W R J, Kremer Hannie (2008). Mid-frequency DFNA8/12 hearing loss caused by a synonymous TECTA mutation that affects an exonic splice enhancer. European Journal of Human Genetics.

[25] Meyer NC (2007). Identification of three novel TECTA mutations in Iranian families with autosomal recessive nonsyndromic hearing impairment at the DFNB21 locus. American Journal of Medical Genetics Part A.

[26] Alasti F (2008). A novel TECTA mutation confirms the recognizable phenotype among autosomal recessive hearing impairment families. International journal of pediatric otorhinolaryngology.

[27] Liu X (2009). Disruption of striated preferentially expressed gene locus leads to dilated cardiomyopathy in mice. Circulation.

[28] Agrawal PB (2014). SPEG interacts with myotubularin, and its deficiency causes centronuclear myopathy with dilated cardiomyopathy. The American Journal of Human Genetics.

[29] Stambas, J. *et al*. (Am Assoc Immnol 2017).

[30] McMahon, M. K., McCulloch, D. & Stambas, J. (Am Assoc Immnol 2016).

[31] Zhou, X., Seim, I. & Gladyshev, V. N. Convergent evolution of marine mammals is associated with distinct substitutions in common genes. *Scientific reports* **5** (2015). 10.1038/srep16550 PMC4637874 26549748

[32] Yang Z (1998). Likelihood ratio tests for detecting positive selection and application to primate lysozyme evolution. Molecular biology and evolution.

[33] Wakil SJ (1989). Fatty acid synthase, a proficient multifunctional enzyme. Biochemistry.

[34] Loftus TM (2000). Reduced food intake and body weight in mice treated with fatty acid synthase inhibitors. Science.

[35] Firth AL (2009). Hypoxia selectively inhibits KCNA5 channels in pulmonary artery smooth muscle cells. Annals of the New York Academy of Sciences.

[36] Platoshyn O (2006). Acute hypoxia selectively inhibits KCNA5 channels in pulmonary artery smooth muscle cells. American Journal of Physiology-Cell Physiology.

[37] Bär E, Whitney PG, Moor K, e Sousa CR, LeibundGut-Landmann S (2014). IL-17 regulates systemic fungal immunity by controlling the functional competence of NK cells. Immunity.

[38] Cypowyj S, Picard C, Marodi L, Casanova JL, Puel A (2012). Immunity to infection in IL‐17‐deficient mice and humans. European journal of immunology.

[39] Reichmuth C, Holt MM, Mulsow J, Sills JM, Southall BL (2013). Comparative assessment of amphibious hearing in pinnipeds. Journal of Comparative Physiology A.

[40] Wartzok D, Ketten DR (1999). Marine mammal sensory systems. Biology of marine mammals.

[41] Verhoeven K (1998). Mutations in the human α-tectorin gene cause autosomal dominant non-syndromic hearing impairment. Nature genetics.

[42] Michalski N, Petit C (2015). Genetics of auditory mechano-electrical transduction. Pflügers Archiv-European Journal of Physiology.

[43] Ishikawa K (2014). A Japanese family showing high-frequency hearing loss with KCNQ4 and TECTA mutations. Acta oto-laryngologica.

[44] Collin RW (2008). Mid-frequency DFNA8/12 hearing loss caused by a synonymous TECTA mutation that affects an exonic splice enhancer. European Journal of Human Genetics.

[45] Moteki H (2012). TECTA mutations in Japanese with mid-frequency hearing loss affected by zona pellucida domain protein secretion. Journal of human genetics.

[46] Dunn OJ (1961). Multiple comparisons among means. Journal of the American Statistical Association.

[47] Davis RW (2014). A review of the multi-level adaptations for maximizing aerobic dive duration in marine mammals: from biochemistry to behavior. Journal of Comparative Physiology B.

[48] Parker J (2013). Genome-wide signatures of convergent evolution in echolocating mammals. Nature.

[49] Marçais G, Kingsford C (2011). A fast, lock-free approach for efficient parallel counting of occurrences of k-mers. Bioinformatics.

[50] Andrews, S. FQC: A quality control tool for high throughput sequence data. *Reference Source* (2010).

[51] Bolger AM, Lohse M, Usadel B (2014). Trimmomatic: a flexible trimmer for Illumina sequence data. Bioinformatics.

[52] O’Connell J (2015). NxTrim: optimized trimming of Illumina mate pair reads. Bioinformatics.

[53] Gnerre S (2011). High-quality draft assemblies of mammalian genomes from massively parallel sequence data. Proceedings of the National Academy of Sciences.

[54] Peng Y, Leung HC, Yiu S-M, Chin FY (2012). IDBA-UD: a de novo assembler for single-cell and metagenomic sequencing data with highly uneven depth. Bioinformatics.

[55] Boetzer M, Henkel CV, Jansen HJ, Butler D, Pirovano W (2010). Scaffolding pre-assembled contigs using SSPACE. Bioinformatics.

[56] Mandric I, Zelikovsky A (2015). ScaffMatch: scaffolding algorithm based on maximum weight matching. Bioinformatics.

[57] Luo R (2012). SOAPdenovo2: an empirically improved memory-efficient short-read de novo assembler. Gigascience.

[58] Smit, A. & Hubley, R. RepeatModeler Open-1.0. *Repeat Masker Website* (2010).

[59] Bao Z, Eddy SR (2002). Automated de novo identification of repeat sequence families in sequenced genomes. Genome research.

[60] Price AL, Jones NC, Pevzner PA (2005). De novo identification of repeat families in large genomes. Bioinformatics.

[61] Benson G (1999). Tandem repeats finder: a program to analyze DNA sequences. Nucleic acids research.

[62] Jurka J (2005). Repbase Update, a database of eukaryotic repetitive elements. Cytogenetic and genome research.

[63] Tarailo‐Graovac, M. & Chen, N. Using RepeatMasker to identify repetitive elements in genomic sequences. *Current protocols in bioinformatics*, 4.10. 11-14.10. 14 (2009). 10.1002/0471250953.bi0410s25 19274634

[64] Consortium, U. UniProt: a hub for protein information. *Nucleic acids research*, gku989 (2014). 10.1093/nar/gku989 PMC4384041 25348405

[65] Altschul SF (1997). Gapped BLAST and PSI-BLAST: a new generation of protein database search programs. Nucleic acids research.

[66] Slater GSC, Birney E (2005). Automated generation of heuristics for biological sequence comparison. BMC bioinformatics.

[67] Stanke M (2006). AUGUSTUS: ab initio prediction of alternative transcripts. Nucleic acids research.

[68] Blanco, E., Parra, G. & Guigó, R. Using geneid to identify genes. *Current protocols in bioinformatics*, 4.3. 1-4.3. 28 (2007). 10.1002/0471250953.bi0403s18 18428791

[69] Majoros WH, Pertea M, Salzberg SL (2004). TigrScan and GlimmerHMM: two open source ab initio eukaryotic gene-finders. Bioinformatics.

[70] Haas BJ (2008). Automated eukaryotic gene structure annotation using EVidenceModeler and the Program to Assemble Spliced Alignments. Genome biology.

[71] Simão FA, Waterhouse RM, Ioannidis P, Kriventseva EV, Zdobnov EM (2015). BUSCO: assessing genome assembly and annotation completeness with single-copy orthologs. Bioinformatics.

[72] Altschul SF, Gish W, Miller W, Myers EW, Lipman DJ (1990). Basic local alignment search tool. Journal of molecular biology.

[73] Boeckmann B (2003). The SWISS-PROT protein knowledgebase and its supplement TrEMBL in 2003. Nucleic acids research.

[74] Zdobnov EM, Apweiler R (2001). InterProScan–an integration platform for the signature-recognition methods in InterPro. Bioinformatics.

[75] Tyner C (2016). The UCSC Genome Browser database: 2017 update. Nucleic acids research.

[76] Li L, Stoeckert CJ, Roos DS (2003). OrthoMCL: identification of ortholog groups for eukaryotic genomes. Genome research.

[77] Larkin MA (2007). Clustal W and Clustal X version 2.0. bioinformatics.

[78] Castresana J (2000). Selection of conserved blocks from multiple alignments for their use in phylogenetic analysis. Molecular biology and evolution.

[79] Keane, T., Naughton, T. & McInerney, J. ModelGenerator: amino acid and nucleotide substitution model selection. *National University of Ireland*, *Maynooth*, *Ireland*, 34 (2004).

[80] Stamatakis A (2006). RAxML-VI-HPC: maximum likelihood-based phylogenetic analyses with thousands of taxa and mixed models. Bioinformatics.

[81] Jones DT, Taylor WR, Thornton JM (1992). The rapid generation of mutation data matrices from protein sequences. Computer applications in the biosciences: CABIOS.

[82] Keane TM, Creevey CJ, Pentony MM, Naughton TJ, Mclnerney JO (2006). Assessment of methods for amino acid matrix selection and their use on empirical data shows that ad hoc assumptions for choice of matrix are not justified. BMC evolutionary biology.

[83] Suyama M, Torrents D, Bork P (2006). PAL2NAL: robust conversion of protein sequence alignments into the corresponding codon alignments. Nucleic acids research.

[84] Yang Z (2007). PAML 4: phylogenetic analysis by maximum likelihood. Molecular biology and evolution.

[85] Zhang J, Nielsen R, Yang Z (2005). Evaluation of an improved branch-site likelihood method for detecting positive selection at the molecular level. Molecular biology and evolution.

[86] Ashkenazy H (2012). FastML: a web server for probabilistic reconstruction of ancestral sequences. Nucleic acids research.

[87] Blanchette M (2004). Aligning multiple genomic sequences with the threaded blockset aligner. Genome research.

[88] Marchler-Bauer A (2014). CDD: NCBI’s conserved domain database. Nucleic acids research.

[89] Flicek P (2011). Ensembl 2012. Nucleic acids research.

[90] Dennis G (2003). DAVID: database for annotation, visualization, and integrated discovery. Genome biology.

