# Supplementary material for: Deciphering the evolutionary signatures of pinnipeds using novel genome sequences: The first genomes of Phoca largha, Callorhinus ursinus, and Eumetopias jubatus
Source: Sci Rep. 2018 Nov 15;8:16877. doi: 10.1038/s41598-018-34758-0 (PMC6237890; doi:10.1038/s41598-018-34758-0)
Supplement: Supplementary file 1 — Supplementary information [file 41598_2018_34758_MOESM1_ESM.docx]

Deciphering the evolutionary signatures of pinnipeds using novel genome sequences: The first genomes of *Phoca largha*, *Callorhinus ursinus*, and *Eumetopias jubatus*

Jung Youn Park^1¶^, Kwondo Kim^2,3¶^, Hawsun Sohn^4^, Hyun Woo Kim^4^, Yong-Rock An^5^, Jung-Ha Kang^1^, Eun-Mi Kim^1^, Woori Kwak^3^, Chul Lee^2^, DongAhn Yoo^2,3^, Jaehoon Jung^3,6^, Samsun Sung^3^, Joon Yoon^2^, Heebal Kim^2,3,6*^

^1^Biotechnology Research Division, National Institute of Fisheries Science, 216 Haean-ro, Gijang-eup, Gijang gun, Busan 46083, Republic of Korea

^2^Interdisciplinary Program in Bioinformatics, Seoul National University, Kwan-ak Gu, Seoul, Republic of Korea

^3^C&K genomics, C-1008, H businesspark, 26, Beobwon-ro 9-gil, Songpa-gu, Seoul, Republic of Korea

^4^Cetacean Research Institute, National Institute of Fisheries Science, 250 Jangsaengpo Gorae-ro, Nam-gu, Ulsan 44780, Republic of Korea

^5^Department of Taxonomy and Systematics, National Marine Biodiversity Institute of Korea, eocheon-gun, Chungcheongnam-do 33662, Republic of Korea

^6^Department of Agricultural Biotechnology, Seoul National University, Kwan-ak Gu, Seoul, Republic of Korea

^¶^These authors contributed equally to this work. Correspondence should be addressed to H.B.K. (heebal@snu.ac.kr)

| **SUPPLEMENTARY INFORMATION** |
| --- |

1. **Supplementary Note……………………………………………………….. 3**
2. **Supplementary Tables**

**Supplementary Table 1…………………………………………………….. 5**

**Supplementary Table 2…………………………………………………….. 6**

**Supplementary Table 3…………………………………………………….. 7**

**Supplementary Table 4…………………………………………………….. 8**

**Supplementary Table 5…………………………………………………….. 9**

**Supplementary Table 6…………………………………………………….. 10**

**Supplementary Table 7…………………………………………………….. 12**

1. **Supplementary Figures**

**Supplementary Figure 1…………………………………………………… 13**

**Supplementary Figure 2…………………………………………………… 14**

**Supplementary Figure 3…………………………………………………… 15**

**Supplementary Figure 4…………………………………………………… 16**

**Supplementary Figure 5…………………………………………………… 17**

**Supplementary Figure 6…………………………………………………… 18**

**Supplementary Figure 7…………………………………………………… 19**

**Supplementary Figure 8…………………………………………………… 20**

**Supplementary Figure 9…………………………………………………… 21**

**Supplementary Figure 10………………………………………………..… 22**

**Supplementary Figure 11………………………………………………..… 23**

**Supplementary Note 1.** General characteristics of the three pinniped species.

**Spotted seal (*Phoca larga*).** The spotted seal is a member of the family *Phocidae*[^1^](#_ENREF_1), and inhabits the North Pacific Ocean including portions of the Beaufort, Chukchi, East Siberian, Bering, Okhotsk, and Yellow Seas, and the East Sea (Sea of Japan in the Japanese literature)[^2^](#_ENREF_2). The body of the spotted seal has short flippers in the anterior and posterior parts. The hind flippers cannot be turned forward under its body for walking on land, so it crawls in contrast to other species of the family *Otariidae*[^3^](#_ENREF_3). It also uses the hind flipper to move in water, in contrast to phocids, which use the front flippers[^3^](#_ENREF_3). The entire body is covered in dense fur and random dark spots[^4^](#_ENREF_4). The spotted seal is is categorized as a species of least concern on the 2017 International Union for Conservation of Nature (IUCN) Red List (<http://www.iucnredlist.org/>).

**Northern fur seal (*Callorhinus ursinus*).** The Northern fur seal is a member of the family *Otariidae* with visible external ear flaps, and is the only living species of the genus *Callorhinus*[^1^](#_ENREF_1). It lives in the North Pacific Ocean, Bering Sea, and Sea of Okhotsk[^5^](#_ENREF_5). This species has a thick fur coat over a portion of the body[^4^](#_ENREF_4). Northern fur seals have extreme sexual dimorphism, particularly in body size[^6^](#_ENREF_6). While northern fur seals are polygynous like all other otariids, a male possesses females in the form of a harem[^5^](#_ENREF_5). The northern fur seal uses the hind flipper to move on land, and the front flippers for swimming, similar to other otariids[^3^](#_ENREF_3). The northern fur seal is categorized as a vulnerable species on the 2017 IUCN Red List (<http://www.iucnredlist.org/>).

Steller sea lion (*Eumetopias jubatus*). The Steller sea lion is a member of the family *Otariidae* that has visible external ear flaps[^1^](#_ENREF_1). The Steller sea lion is the largest otariid species and the only member of the genus *Eumetopias*^[7](#_ENREF_7)^. The range of the Steller sea lion extends from the Kuril Islands and the Sea of Okhotsk to the Gulf of Alaska[^7^](#_ENREF_7). The Steller sea lion exhibits marked sexual dimorphism in terms of body size[^7^](#_ENREF_7). Although like the Northern fur seal it is polygynous, instead of forming a harem male Steller sea lions control a spatial area within which females freely move[^8^](#_ENREF_8). The Steller sea lion is categorized as a near-threatened species on the 2017 IUCN Red List (<http://www.iucnredlist.org/>).

**Supplementary Table 1**. Summary of assembly statistics (>2000bp)

|  | **Spotted Seal** | **Northern Fur seal** | **Steller Sea Lion** |
| --- | --- | --- | --- |
| **Size (Haploid)** | 2.26 Gb | 2.37 Gb | 2.36 Gb |
| **GC level** | 41.36% | 41.39% | 40.98% |
| **No. scaffolds** | 50053 | 69688 | 17807 |
| **N50 of scaffolds (bp)** | 87534 | 67633 | 331138 |
| **N bases in scaffolds (%)** | 0.2 Mb (0.01%) | 0.6 Mb (0.03%) | 21 Mb (0.90%) |
| **Longest (shortest) scaffolds (bp)** | 732428 | 606905 (2000) | 2503745 (2000) |
| **Average scaffold length (bp)** | 45467.84 | 33959.64 | 132798.99 |

**Supplementary Table 2.** Summary of genome assessment results

|  | **Spotted Seal** | **Norther Fur Seal** | **Stella Sea Lion** |
| --- | --- | --- | --- |
| **Overall remapping rate** | 98.73% | 98.74% | 98.24% |
| **Complete single-copy BUSCOs** | 3652 (89.0%) | 3101 (75.6%) | 3169 (77.2%) |
| **Complete duplicate BUSCOs** | 49 (1.2%) | 39 (1.0%) | 28 (0.7%) |
| **Fragmented BUSCOs** | 241 (5.9%) | 641 (15.6%) | 613 (14.9%) |
| **Missing BUSCOs** | 162 (3.9%) | 323 (7.8%) | 294 (7.2%) |

**Supplementary Table 3.** Summary statistics for repeat elements

|  | **Spotted Seal** | | **Norther Fur Seal** | | **Stella Sea Lion** | |
| --- | --- | --- | --- | --- | --- | --- |
| **Repeat element** | **No. element** | **Length (%)** | **No. element** | **Length bp (%)** | **No. element** | **Length (%)** |
| **SINE** | 1,036,137 | 141,553,944 (5.58%) | 1,129,582 | 161,422,763 (5.71%) | 851,418 | 133,941,433 (5.07%) |
| **LINE** | 2,214,162 | 585,943,176 (23.08%) | 3,011,201 | 743,144,050 (26.28%) | 2,377,806 | 660,209,680 (25.01%) |
| **LTR element** | 197,943 | 82,674,816 (3.26%) | 354,083 | 99,715,643 (3.53%) | 158,944 | 69,491,321 (2.63%) |
| **DNA element** | 125,810 | 35,396,811 (1.39%) | 142,116 | 36,834,655 (1.30%) | 97,051 | 29,844,098 (1.13%) |
| **Small RNA** | 975,134 | 134,338,873 (5.29%) | 1,066,608 | 153,715,199 (5.44%) | 801,275 | 128,404,897 (4.86%) |
| **Satellites** | 2,177 | 697,132 (0.03%) | 12,501 | 2,277,985 (0.08%) | 1,750 | 223,507 (0.01%) |
| **Simple repeat** | 767,846 | 35,008,073 (1.38%) | 886,899 | 41,726,927 (1.48%) | 819,929 | 39,293,888 (1.49%) |
| **Low complexity** | 106,255 | 5,545,681 (0.22%) | 133,029 | 6,805,611 (0.24%) | 108,031 | 5,560,112 (0.21%) |
| **Unclassified** | 145,816 | 24,567,042 (0.97%) | 371,817 | 52,945,392 (1.87%) | 33,385 | 6,259,843 (0.24%) |

**Supplementary Table 4.** Summary statistics of gene prediction results

| **Species** | **Element** | **No. of elements** | **Average length** | **Count per gene** | **Total length** | **Genome coverage** |
| --- | --- | --- | --- | --- | --- | --- |
| **Spotted Seal** | **Gene** | 33,988 | 13,012.64 | - | 442,273,447 | 0.17 |
|  | **Exon** | 177,734 | 194.80 | 5.23 | 34,622,532 | 0.01 |
|  | **Intron** | 143,746 | 2,835.91 | 4.23 | 407,650,915 | 0.16 |
| **Northern Fur Seal** | **Gene** | 32,740 | 12,642.54 | - | 413,916,607 | 0.15 |
|  | **Exon** | 172,034 | 192.00 | 5.25 | 33,030,109 | 0.01 |
|  | **Intron** | 139,294 | 2,734.41 | 4.25 | 380,886,498 | 0.13 |
| **Steller Sea Lion** | **Gene** | 28,081 | 21,049.79 | - | 591,099,025 | 0.22 |
|  | **Exon** | 180,207 | 209.79 | 6.42 | 37,805,425 | 0.01 |
|  | **Intron** | 152,126 | 3,637.07 | 5.42 | 553,293,600 | 0.21 |

**Supplementary Table 5.** Summary of functional annotation results

|  |  | **Spotted Seal** | | **Northern Fur Seal** | | **Stella Sea Lion** | |
| --- | --- | --- | --- | --- | --- | --- | --- |
| **Annotated** | **InterPro** | 23,890 | 70.29% | 23,829 | 72.78% | 21,310 | 75.89% |
|  | **GO** | 18,368 | 54.04% | 18,590 | 56.78% | 17,045 | 60.70% |
|  | **SwissProt** | 31,713 | 93.31% | 30,807 | 94.10% | 25,905 | 92.25% |
|  | **TrEMBL** | 31,462 | 92.57% | 30,401 | 92.86% | 25,664 | 91.39% |
| **Not annotated** |  | 1,922 | 5.65% | 1,682 | 5.14% | 1,962 | 6.99% |
| **Total** |  | 33,988 |  | 32,740 |  | 28,081 |  |

**Supplementary Table 6.** Gene ontology (GO) enrichment analysis of pinnipeds specific gene families (P-value < 0.05)

| **ID** | **GO Term** | **Count** | **%** | **PValue** | **Pop Hits** | **Fold Enrichment** |
| --- | --- | --- | --- | --- | --- | --- |
| GO:0032024 | positive regulation of insulin secretion | 8 | 0.448 | 0.004 | 41 | 3.929 |
| GO:0006366 | transcription from RNA polymerase II promoter | 40 | 2.241 | 0.005 | 513 | 1.570 |
| GO:0019985 | translesion synthesis | 7 | 0.392 | 0.008 | 36 | 3.915 |
| GO:0045944 | positive regulation of transcription from RNA polymerase II promoter | 66 | 3.697 | 0.010 | 981 | 1.355 |
| GO:0035904 | aorta development | 5 | 0.280 | 0.011 | 18 | 5.593 |
| GO:0016126 | sterol biosynthetic process | 4 | 0.224 | 0.011 | 10 | 8.054 |
| GO:0070987 | error-free translesion synthesis | 5 | 0.280 | 0.013 | 19 | 5.298 |
| GO:0042276 | error-prone translesion synthesis | 5 | 0.280 | 0.013 | 19 | 5.298 |
| GO:0007005 | mitochondrion organization | 10 | 0.560 | 0.014 | 77 | 2.615 |
| GO:0002244 | hematopoietic progenitor cell differentiation | 9 | 0.504 | 0.016 | 66 | 2.746 |
| GO:0048538 | thymus development | 7 | 0.392 | 0.019 | 43 | 3.278 |
| GO:0003279 | cardiac septum development | 4 | 0.224 | 0.019 | 12 | 6.711 |
| GO:0032228 | regulation of synaptic transmission, GABAergic | 4 | 0.224 | 0.019 | 12 | 6.711 |
| GO:0000722 | telomere maintenance via recombination | 6 | 0.336 | 0.020 | 32 | 3.775 |
| GO:1901796 | regulation of signal transduction by p53 class mediator | 13 | 0.728 | 0.020 | 124 | 2.111 |
| GO:0009792 | embryo development ending in birth or egg hatching | 3 | 0.168 | 0.022 | 5 | 12.081 |
| GO:1903232 | melanosome assembly | 3 | 0.168 | 0.022 | 5 | 12.081 |
| GO:0006486 | protein glycosylation | 12 | 0.672 | 0.025 | 113 | 2.138 |
| GO:0006297 | nucleotide-excision repair, DNA gap filling | 5 | 0.280 | 0.029 | 24 | 4.195 |
| GO:0006974 | cellular response to DNA damage stimulus | 18 | 1.008 | 0.030 | 208 | 1.742 |
| GO:0000122 | negative regulation of transcription from RNA polymerase II promoter | 48 | 2.689 | 0.032 | 720 | 1.342 |
| GO:0006273 | lagging strand elongation | 3 | 0.168 | 0.032 | 6 | 10.067 |
| GO:0060976 | coronary vasculature development | 5 | 0.280 | 0.033 | 25 | 4.027 |
| GO:0033683 | nucleotide-excision repair, DNA incision | 6 | 0.336 | 0.038 | 38 | 3.179 |
| GO:0034599 | cellular response to oxidative stress | 8 | 0.448 | 0.039 | 64 | 2.517 |
| GO:0051593 | response to folic acid | 3 | 0.168 | 0.044 | 7 | 8.629 |
| GO:1900264 | positive regulation of DNA-directed DNA polymerase activity | 3 | 0.168 | 0.044 | 7 | 8.629 |
| GO:0006260 | DNA replication | 14 | 0.784 | 0.045 | 155 | 1.819 |
| GO:0030326 | embryonic limb morphogenesis | 6 | 0.336 | 0.046 | 40 | 3.020 |
| GO:0006470 | protein dephosphorylation | 12 | 0.672 | 0.049 | 126 | 1.918 |
| GO:0048663 | neuron fate commitment | 4 | 0.224 | 0.049 | 17 | 4.737 |

**Supplementary Table 7**. Summary of sequencing data

| **Species** | **Library name** | **Library type** | **Insert size** | **Platform** | **Read length** | **No. reads** | **Total bp** |
| --- | --- | --- | --- | --- | --- | --- | --- |
| Steller sea lion | 350bp | Paired-end | 350 | Nextseq500 | 150 | 1,278,234,364 | 191,735,154,600 |
|  | 700bp | Paired-end | 700 | Nextseq500 | 150 | 899,461,340 | 134,919,201,000 |
|  | 3k | Mate-pair | 3000 | Nextseq500 | 151 | 421,988,284 | 63,720,230,884 |
|  | 9k | Mate-pair | 9000 | Nextseq500 | 151 | 352,293,972 | 53,196,389,772 |
|  | 40k | Mate-pair | 40000 | Nextseq500 | 151 | 278,537,902 | 42,059,223,202 |
| **Total** |  |  |  |  |  | 3,230,515,862 | 485,630,199,458 |
| Spotted Seal | 350bp | Paired-end | 350 | Nextseq500 | 150 | 1,365,397,528 | 204,809,629,200 |
|  | 700bp | Paired-end | 700 | Nextseq500 | 150 | 1,009,079,390 | 151,361,908,500 |
| **Total** |  |  |  |  |  | 2,374,476,918 | 356,171,537,700 |
| Northern Fur Seal | 350bp | Paired-end | 350 | Nextseq500 | 150 | 1,011,695,208 | 151,754,281,200 |
|  | 700bp | Paired-end | 700 | Nextseq500 | 150 | 1,807,129,054 | 271,069,358,100 |
| **Total** |  |  |  |  |  | 2,818,824,262 | 422,823,639,300 |

Supplementary Figure 1. Examples of (a) *Phoca largha;* Spotted seal, (b) *Callorhinus ursinus;* Northern fur seal, and (c) *Eumetopias jubatus;* Steller sea lion.

**
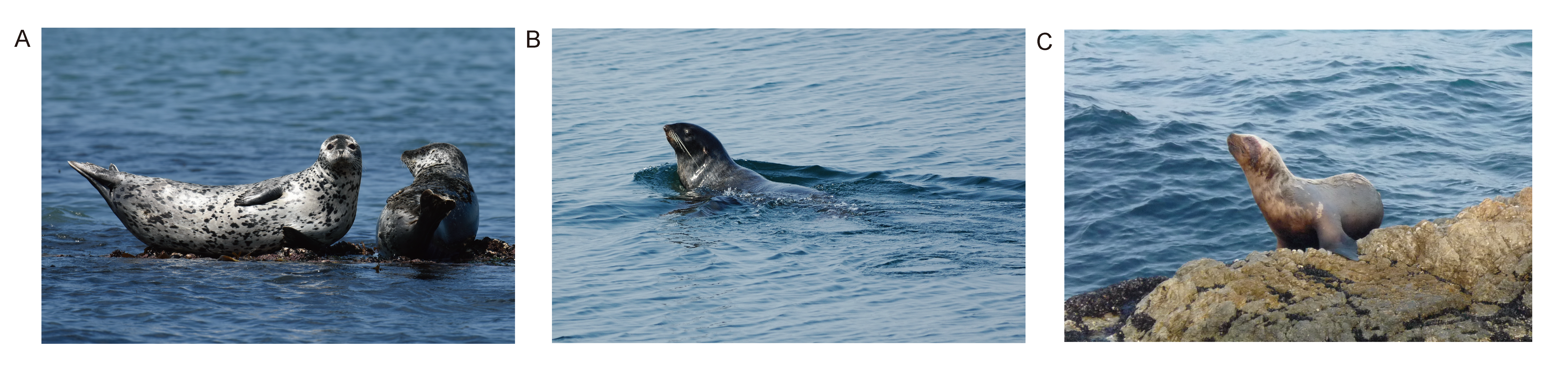
**


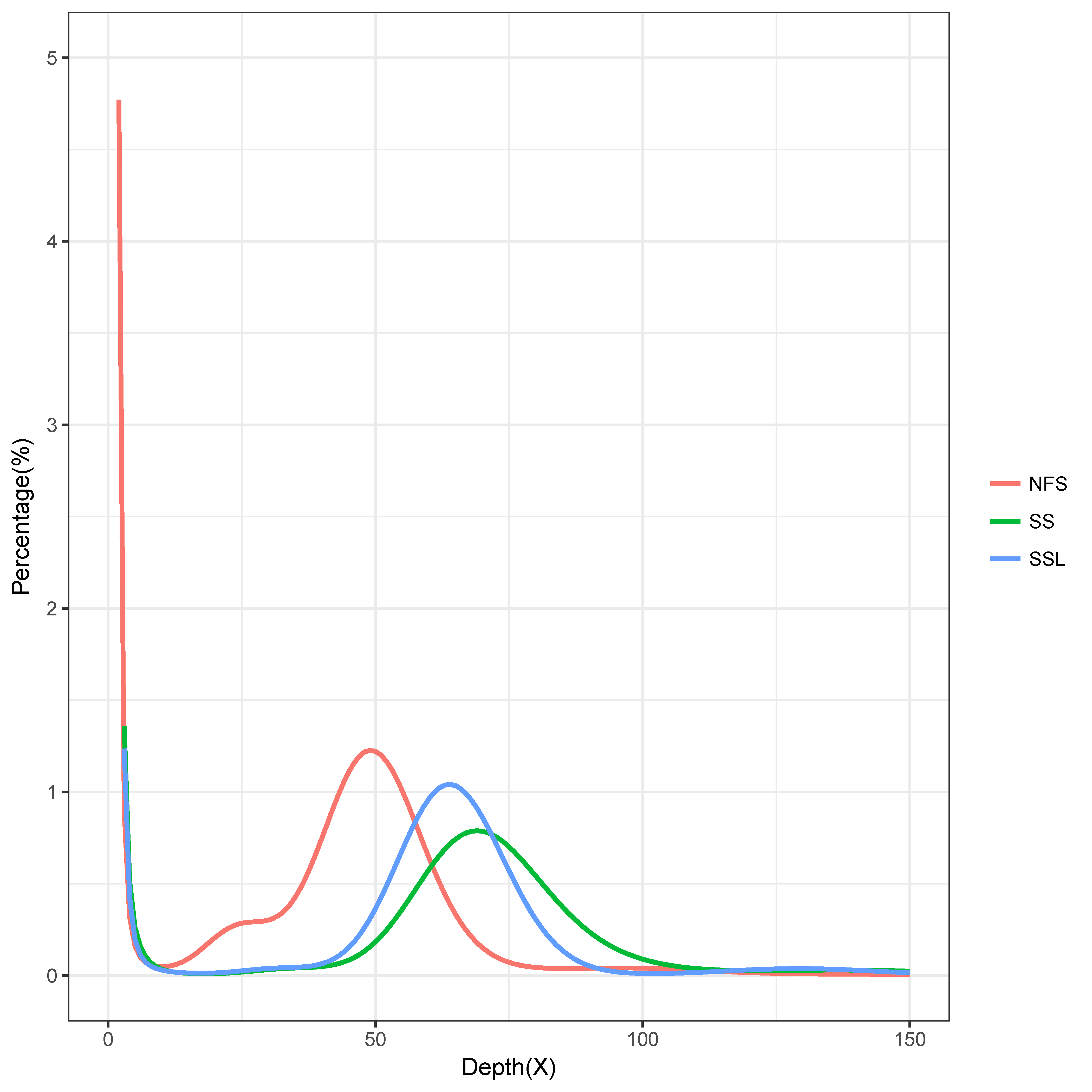
**Supplementary Figure 2.** Estimation of three pinnipeds’ genome sizes based on 19-mer

**Supplementary Figure 3.** Distribution of GC content for the pinnipeds genomes

**
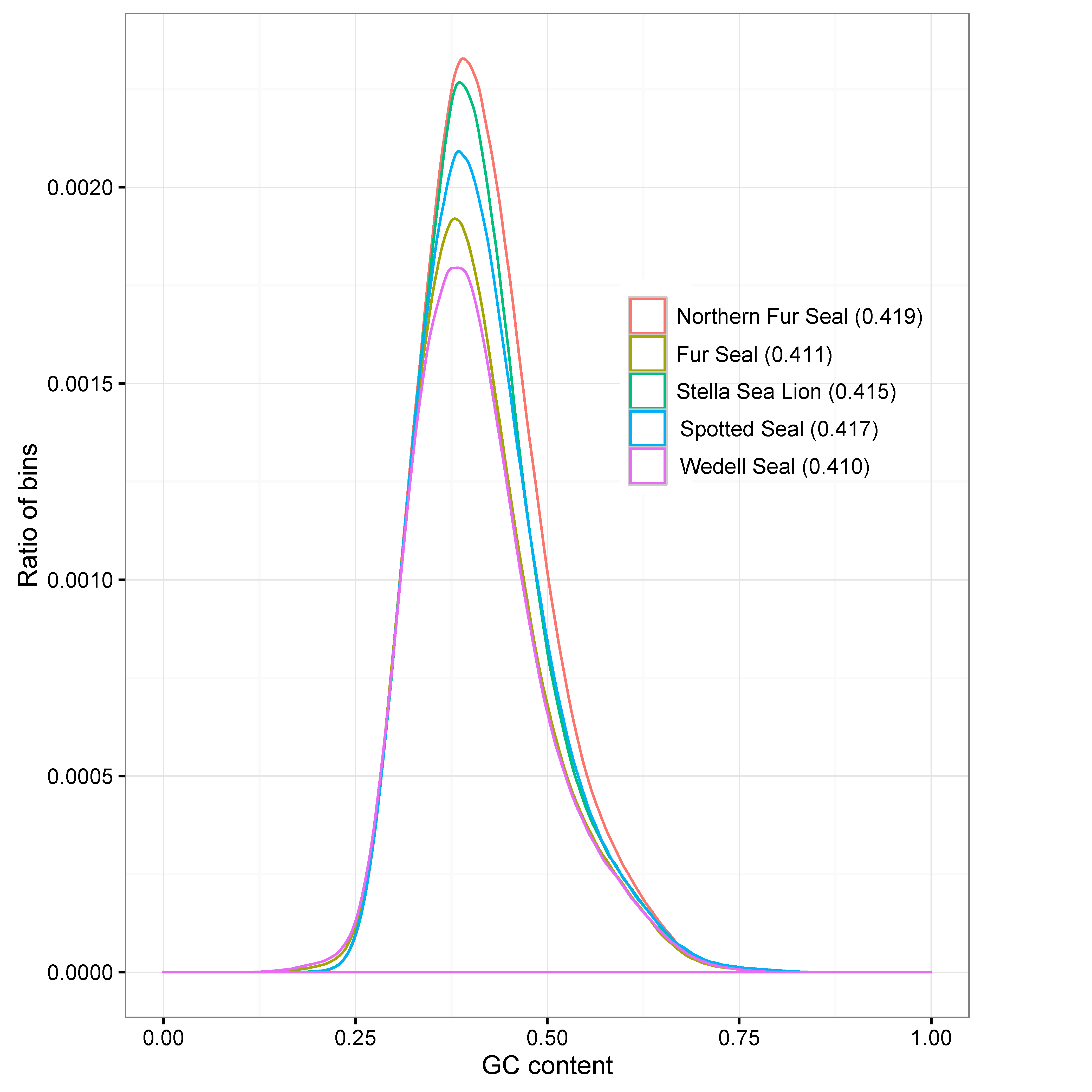
**

**Supplementary Figure 4.** Gene family expansion or contraction across 6 marine mammals


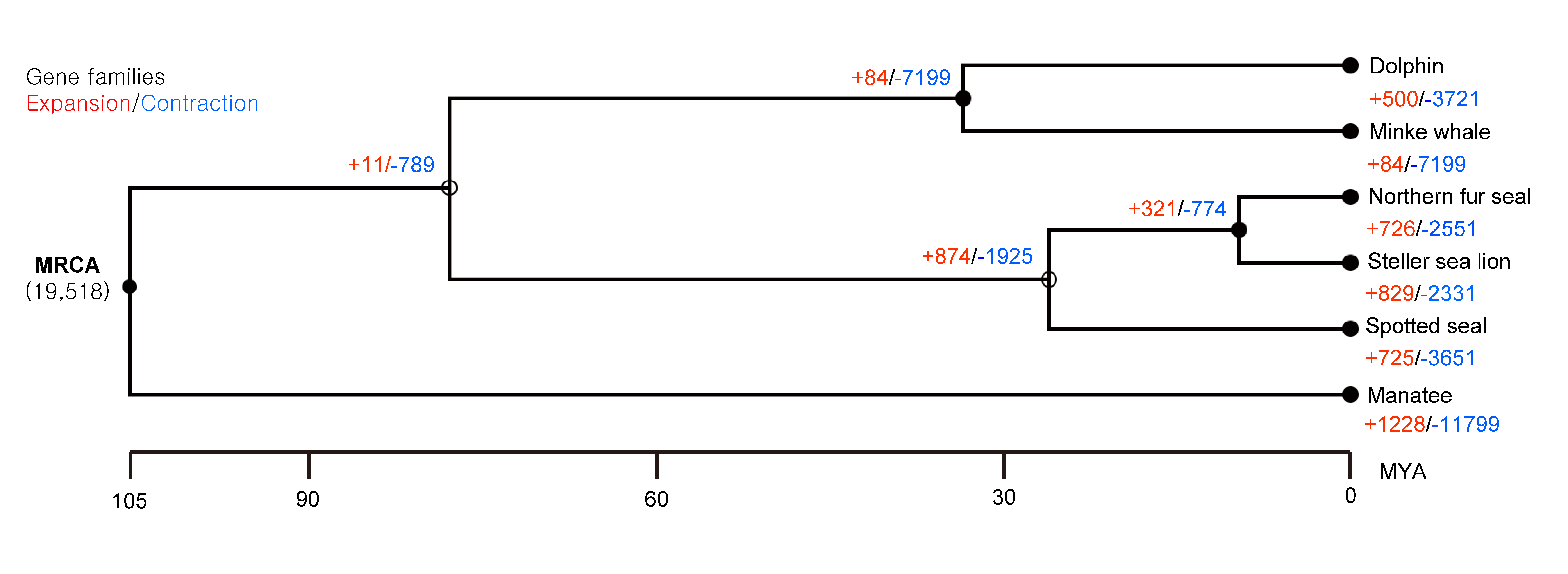


**Supplementary Figure 5.** Amino acid sequences across 100 vertebrates at the site with rapid evolution or substitution unique to three pinnipeds (Spotted seal, Northern fur seal, and Steller sea lion). Sites at *TECTA, FASN, KCNA5 and IL17RA* are shown. ** indicates substitutions unique to pinnipeds. * indicates substitution unique to cetaceans + sirenians.


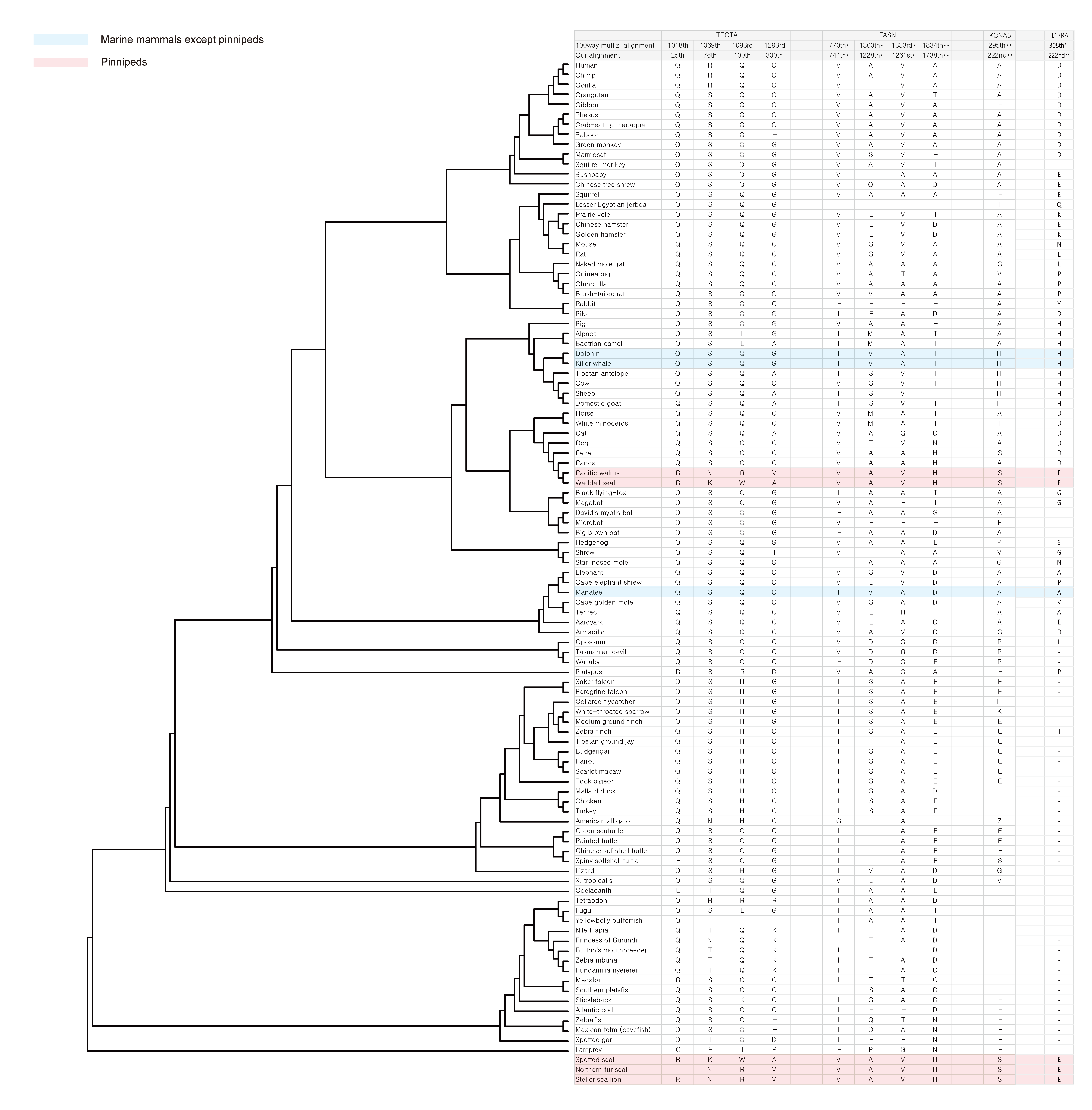


**Supplementary Figure 6.** Number of parallel and unique substitutions across marine mammal clades.

**
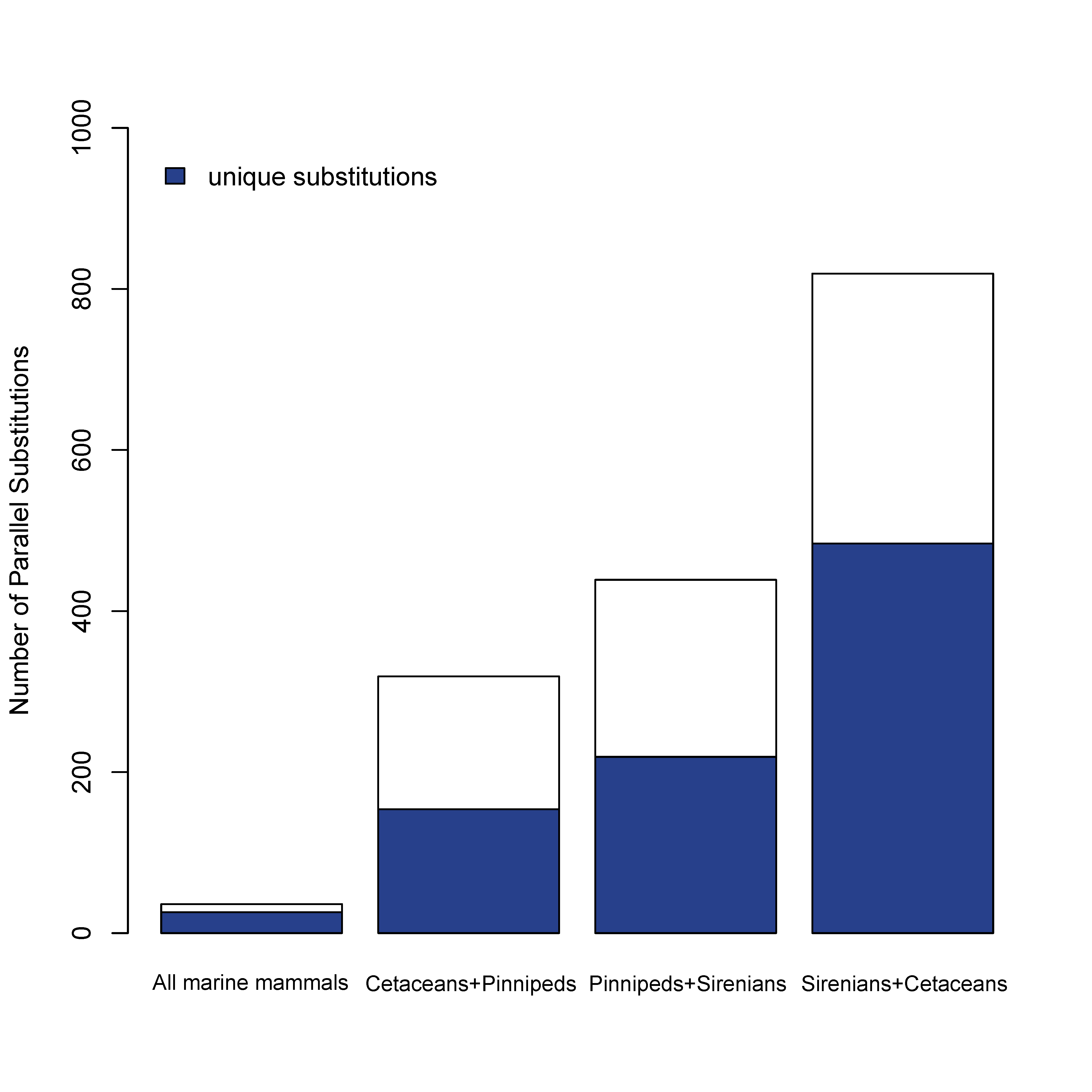
**

**Supplementary Figure 7.** Percentage of unique substitutions in total substitutions for all pairwise comparisons among all 12 species in the phylogeny. The dashed line indicates the average.

**
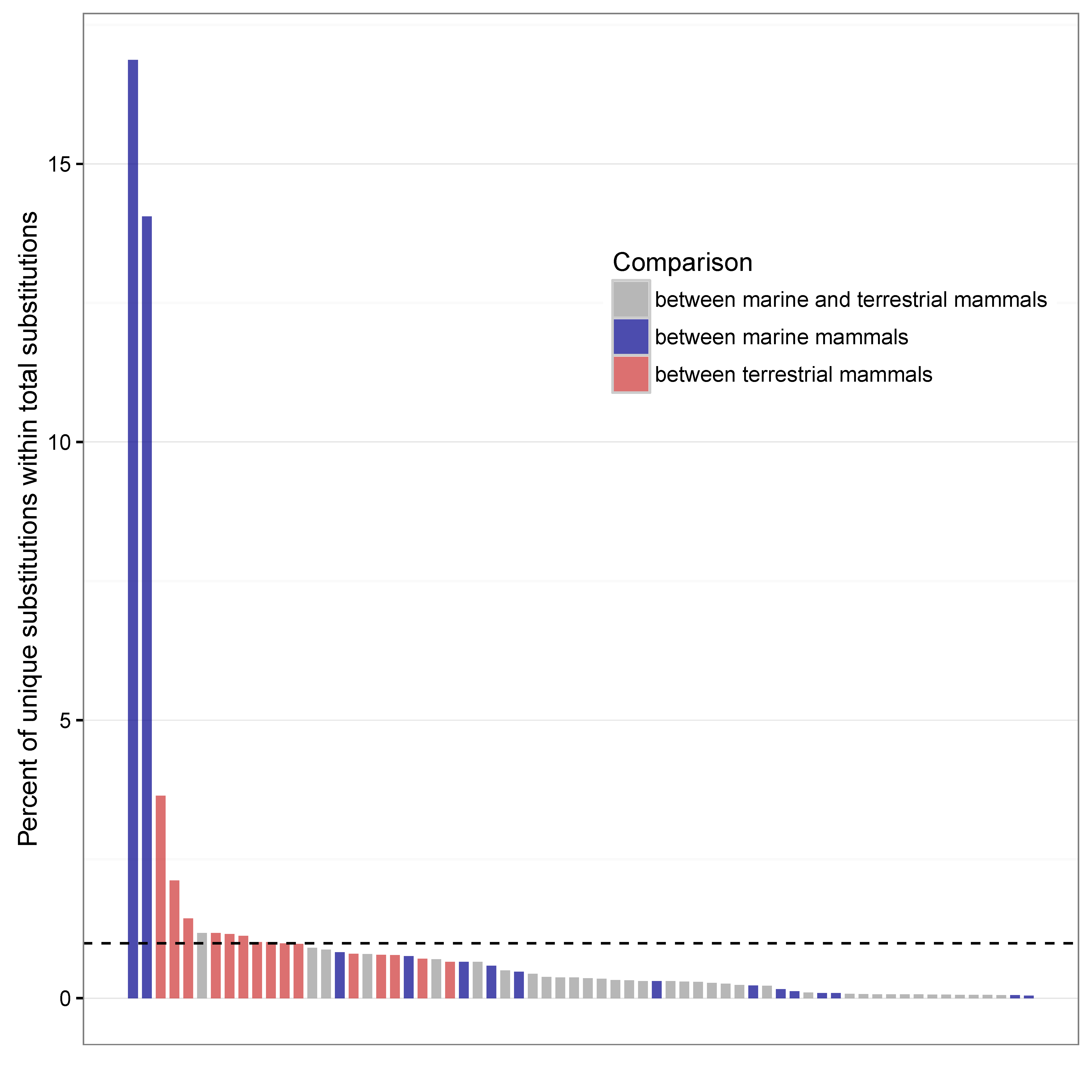
**

**Supplementary Figure 8.** Percentage of unique substitutions in parallel substitutions for all pairwise comparisons among all 12 species in the phylogeny. The dashed line indicates the average

**
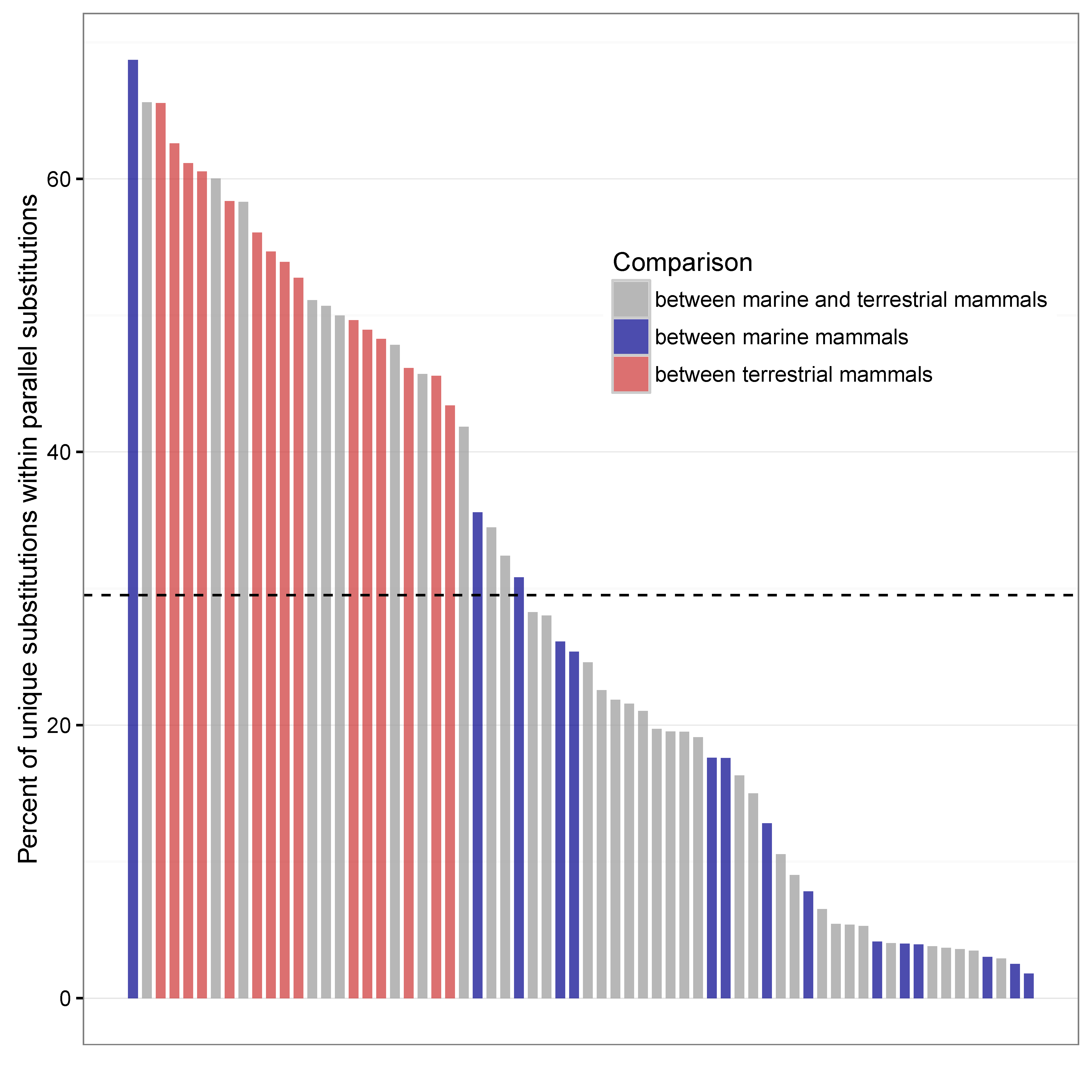
**

**Supplementary Figure 9.** Convergent evolution at multiple levels. Similar phenotypes can evolve at: (a) molecular; (b) gene; (c) phenotypic level^9^. We have focused e on the gene-level convergence (b) in this study.


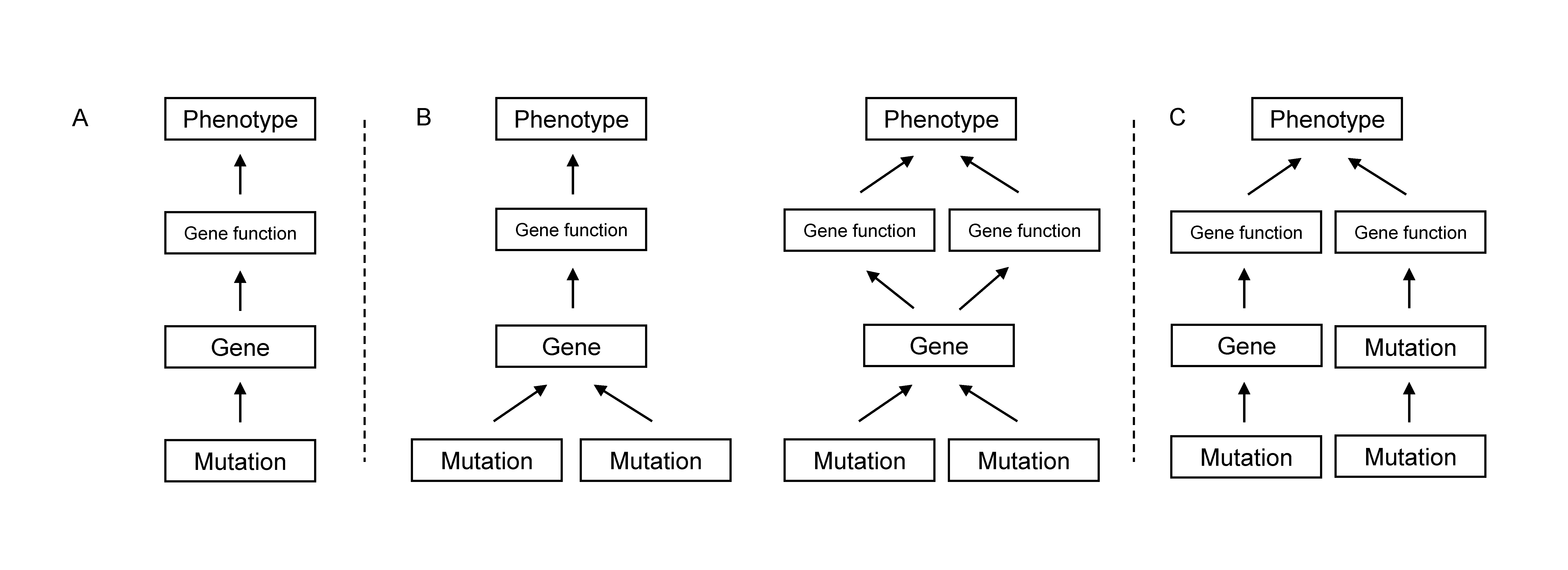


**Supplementary Figure 10.** Foreground branches used in (a) branch-site model, and (b) branch model. Red line indicates foreground branches in each analyses.


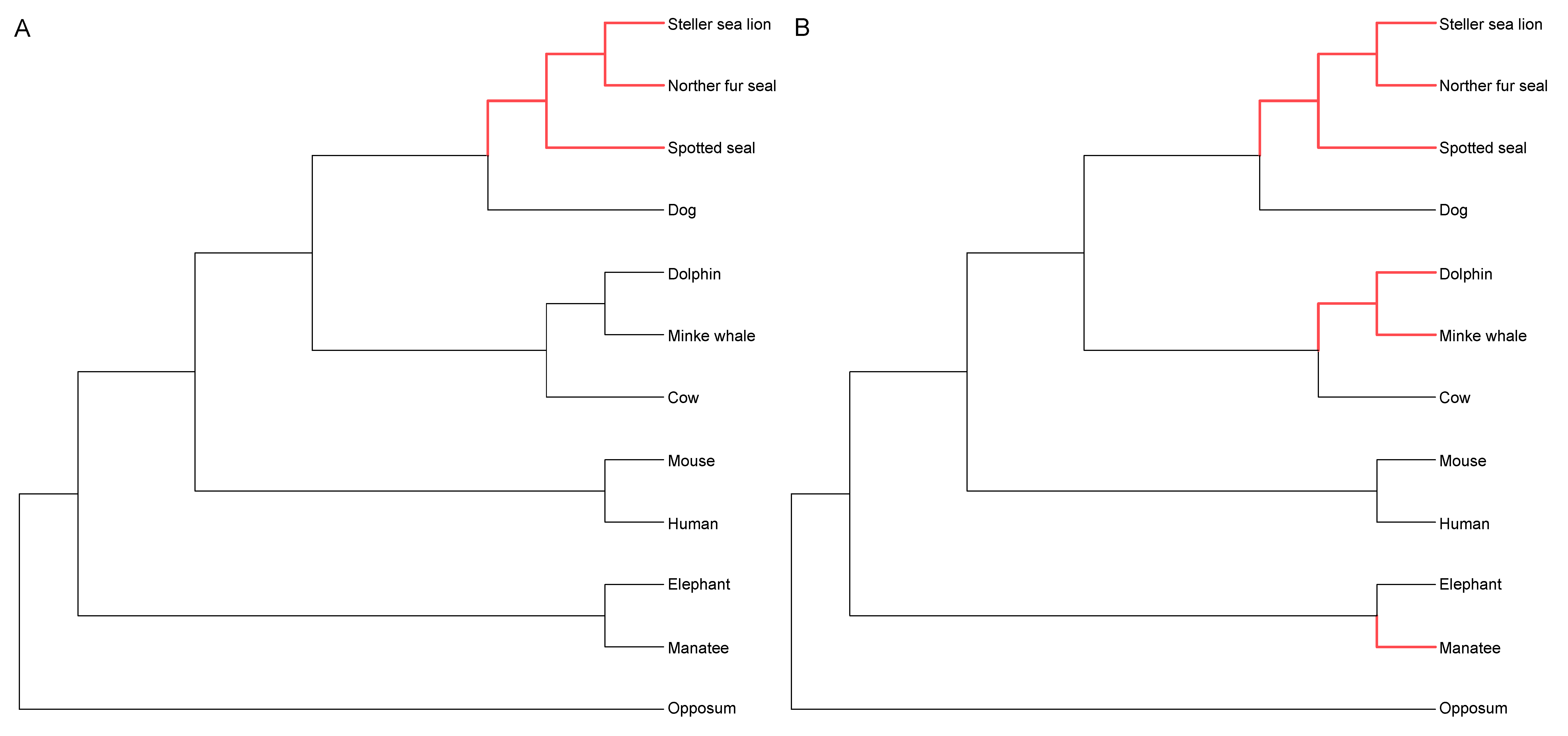


**Supplementary Figure 11.** Intersect between the positively selected genes from branch model analysis and branch-site model analysis for all marine mammal’s branches with continuous p-value cutoffs. Red line indicates the number of positively selected genes from branch model analysis and turquoise line indicates the number of positively selected genes detected in both of branch model and branch-site model analysis.


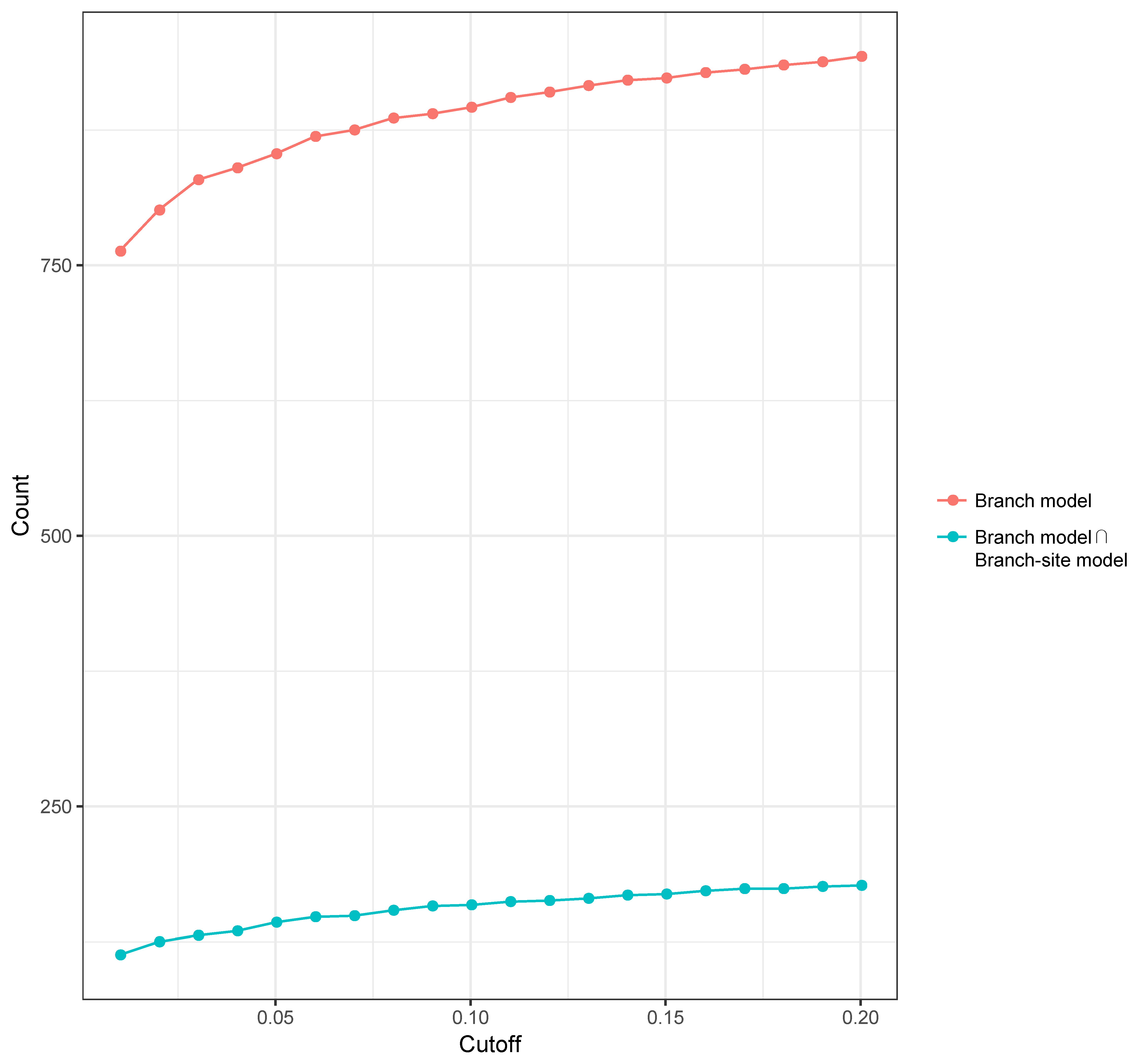


**References**

1 Berta, A. Pinnipedia, overview. *J. Zool* **83**, 1525-1531 (2002).

2 Rugh, D. J., Shelden, K. E. & Withrow, D. E. Spotted seals, Phoca largha, in Alaska. *Marine Fisheries Review* **59**, 1-18 (1997).

3 Riedman, M. *The pinnipeds: seals, sea lions, and walruses*. (Univ of California Press, 1990).

4 Wynne, K. & Folkens, P. Guide to marine mammals of Alaska. *MAB (USA)* (1992).

5 Gentry, R. L. *Behavior and ecology of the northern fur seal*. (Princeton University Press, 1998).

6 Lindenfors, P., Tullberg, B. S. & Biuw, M. Phylogenetic analyses of sexual selection and sexual size dimorphism in pinnipeds. *Behavioral Ecology and Sociobiology* **52**, 188-193 (2002).

7 Loughlin, T. R. The Steller sea lion: a declining species. *Biosphere Conservation* **1**, 91-98 (1998).

8 Gentry, R. L. Social behavior of the Steller sea lion. *Ph. D. dissertation. Univ. Calif.* (1970).

9 Manceau, M., Domingues, V. S., Linnen, C. R., Rosenblum, E. B. & Hoekstra, H. E. Convergence in pigmentation at multiple levels: mutations, genes and function. *Philosophical Transactions of the Royal Society of London B: Biological Sciences* **365**, 2439-2450 (2010).
